# Expression and function of nuclear receptor coactivator 4 isoforms in transformed endometriotic and malignant ovarian cells

**DOI:** 10.18632/oncotarget.23747

**Published:** 2017-12-28

**Authors:** Stephanie Rockfield, Idhaliz Flores, Meera Nanjundan

**Affiliations:** ^1^ Department of Cell Biology, Microbiology, and Molecular Biology, University of South Florida, Tampa, FL, USA; ^2^ Department of Basic Sciences-Microbiology, Ponce Health Sciences University and School of Medicine, Ponce Research Institute, Ponce, Puerto Rico

**Keywords:** NCOA4, ferritinophagy, epithelial ovarian cancer, iron, cell survival

## Abstract

Iron is proposed to contribute to the transition from endometriosis to specific subtypes of ovarian cancers (OVCAs). Regulation of intracellular iron occurs via a ferritinophagic process involving NCOA4 (Nuclear Receptor Coactivator 4), represented by two major isoforms (NCOA4α and NCOA4β), whose contribution to ovarian cancer biology remains uninvestigated. We thus generated transformed endometriotic cells (via HRAS^V12A^, c-MYC^T58A^, and p53 inactivation) whose migratory potential was increased in response to conditioned media from senescent endometriotic cells. We identified elevated NCOA4 mRNA in transformed endometriotic cells (relative to non-transformed). Knockdown of NCOA4 increased ferritin heavy chain (FTH1) and p21 protein which was accompanied by reduced cell survival while NCOA4β overexpression reduced colony formation. NCOA4α and NCOA4β mRNA were elevated in malignant versus non-malignant gynecological cells; NCOA4α protein was increased in the assessed malignant cell lines as well as in a series of OVCA subtypes (relative to normal adjacent tissues). Further, NCOA4 protein expression was regulated in a proteasome- and autophagy-independent manner. Collectively, our results implicate NCOA4 in ovarian cancer biology in which it could be involved in the transition from precursors to OVCA.

## INTRODUCTION

Ovarian cancer (OVCA) is one of the deadliest gynecological cancers in women [1]. Precursors to OVCA include endometriotic lesions (proposed to transition to clear cell/endometrioid) as well as fallopian tube fimbriae and/or ovarian surface epithelium (proposed to transition to serous) [2]. The mechanisms underlying the transition of these precursors, such as endometriosis, to OVCAs remain unclear. Evidence implicates an array of mutations and genomic aberrations that contribute to OVCA development [3]. These include the (a) c-MYC proto-oncogene, located at 8q24.21, which is frequently amplified in >20% of OVCAs [3], (b) p53 tumor suppressor, located at 17p13.1, which is mutated in ∼95% of OVCAs [3], and (c) KRAS proto-oncogene, located at 12p12.1, which is amplified in >10% of OVCA patients [3]. In addition, the HRAS proto-oncogene, located at 11p15.1, is frequently activated due to upstream alterations in signaling pathways; mutations in its gene have been rarely identified [4]. There are reports that alterations in the above described genes can be mediated by reactive oxygen species (ROS) [5]. It is suggested that ROS may be generated by high levels of redox-active iron (mM levels) in endometriomas [6] as well as in the fallopian tube [7]. Indeed, as a result of its ability to undergo Fenton reactions, iron is considered to be highly mutagenic [8, 9]. Furthermore, increased DNA damage is noted in fallopian tube secretory epithelial cells in response to transferrin [10] and cellular treatment with iron leads to their increased proliferative capacity [11]. Nonetheless, the exact role of iron in mediating OVCA pathogenesis requires clarification. Iron levels need to be balanced appropriately within the cell; excess iron is frequently stored in ferritin as a storage complex (for a comprehensive review on iron regulation, see [12]). When iron is required by the cell, it may be released via the process of ferritinophagy, which is mediated by Nuclear Receptor Coactivator 4 (NCOA4) [13] leading to increased redox-active iron that may be utilized for regulation of enzymatic activities which requires formation of iron-sulfur clusters [14]. Two well-studied NCOA4 isoforms (NCOA4α and NCOA4β) have been functionally assessed in breast and prostate cancers [15–18]. Specifically, NCOA4α inhibits proliferation in prostate cancer whereas NCOA4β increases proliferation in both prostate and breast cancer cells [17, 18]. Thus far, there is only limited data describing increased NCOA4 transcripts in ovarian carcinomas [19]; thus, the contribution of NCOA4 to the progression of OVCA has not yet been thoroughly investigated.

Herein, we successfully developed a transformed endometriotic cell line via overexpression of HRAS^V12A^ and c-MYC^T58A^ with p53 inactivation. These cells were able to overcome cellular senescence and displayed altered mRNA expression of genes involved in the EMT and iron pathways, notably NCOA4. Knockdown of this nuclear receptor coactivator in normal/benign and malignant gynecological cells resulted in reduced cell survival. In malignant gynecological cells compared to non-malignant, we identified increased mRNA transcripts for two NCOA4 isoforms (NCOA4α and NCOA4β) as well as increased NCOA4α protein. We additionally identified increased NCOA4 expression in ovarian tumors (serous, serous papillary, and mucinous) compared to adjacent normal tissues via tissue microarray analyses. Together, our results demonstrate that NCOA4 expression is elevated in transformed endometriotic cells (relative to non-transformed endometriotic cells) as well as malignant OVCA cells (relative to non-malignant cells) and furthermore, this molecule appears to regulate survival of these gynecological cells.

## RESULTS

### Characterization of transformed endometriotic cells and their increased migratory response to conditioned media from primary senescent endometriotic cells

**Figure 1:**
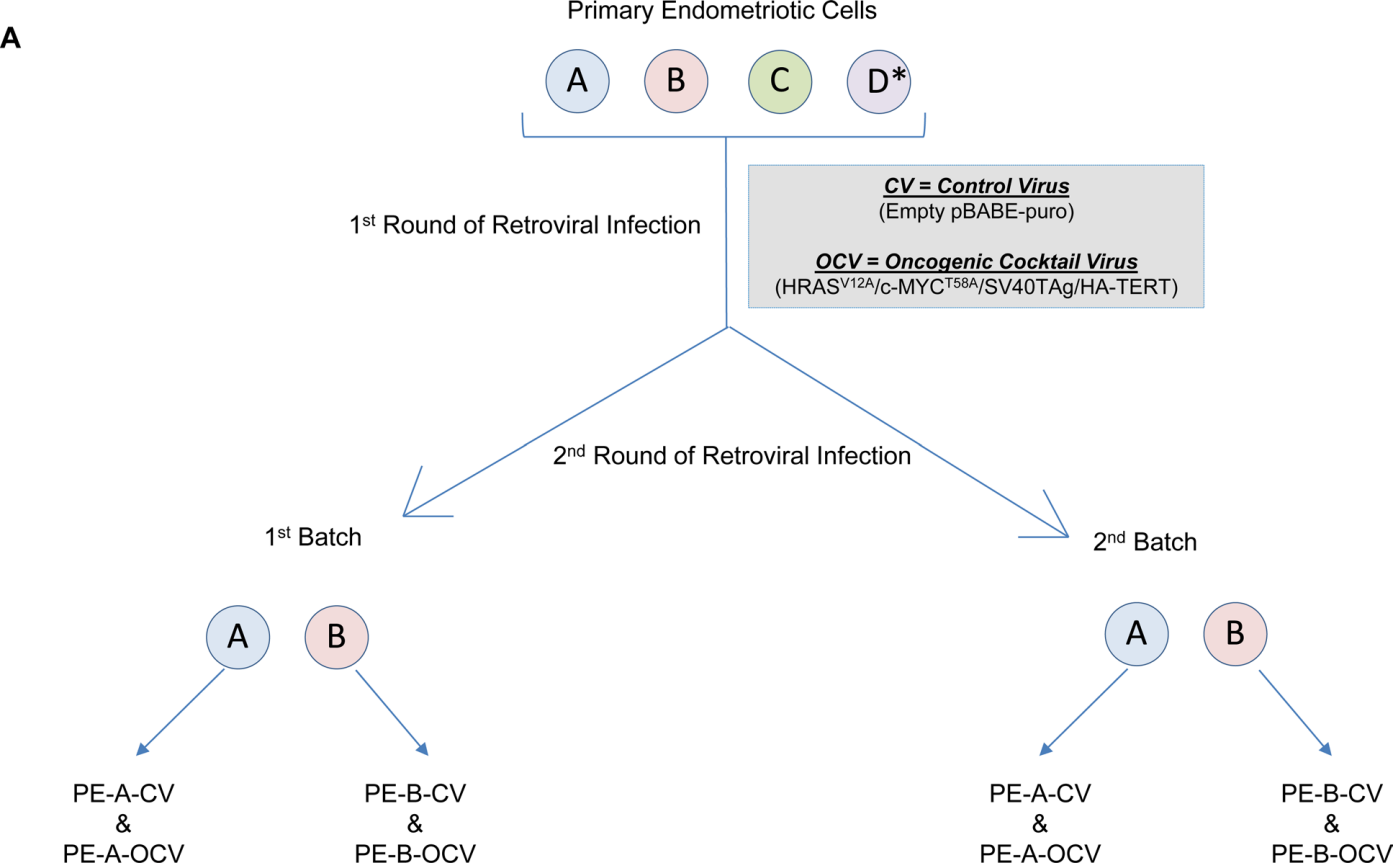

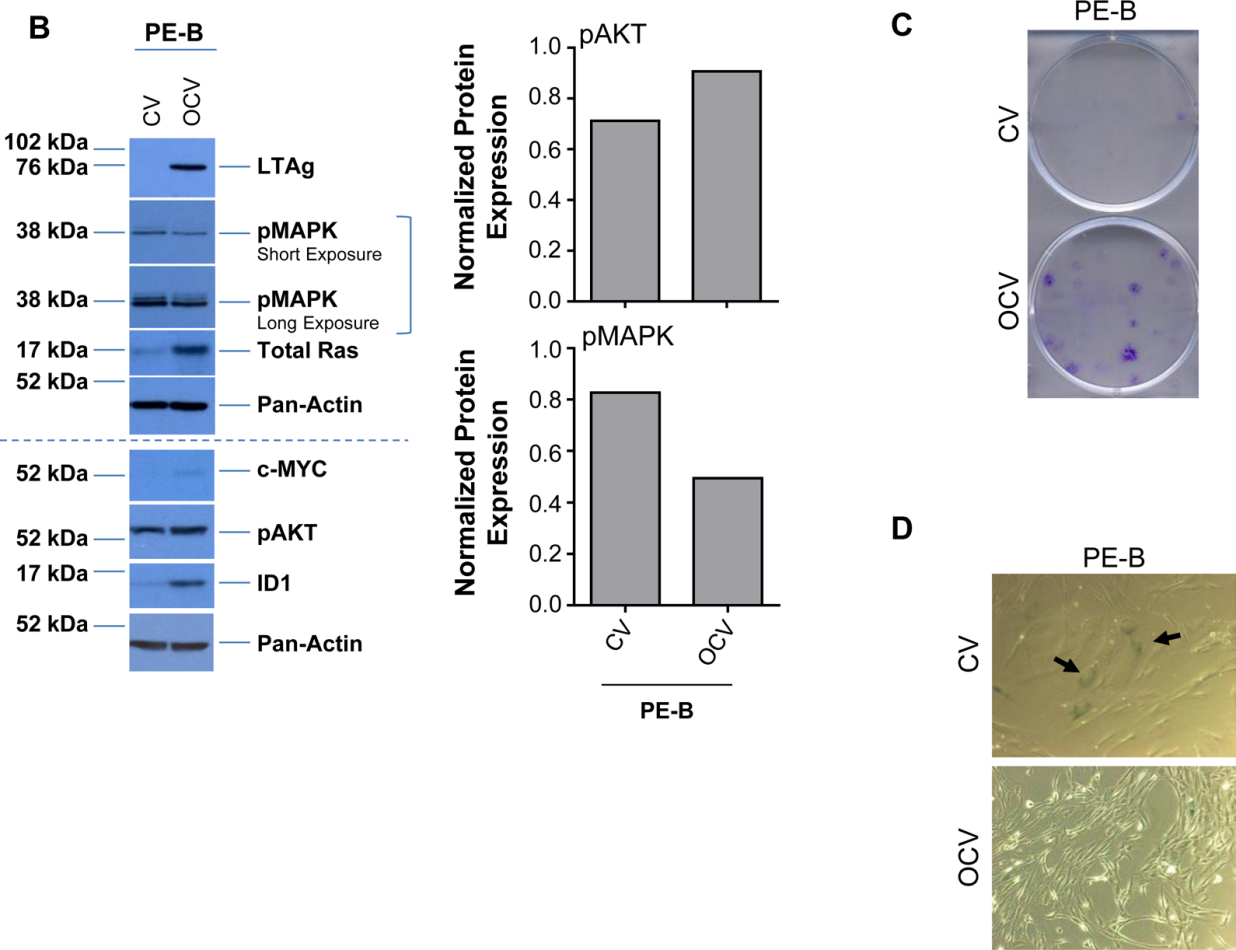

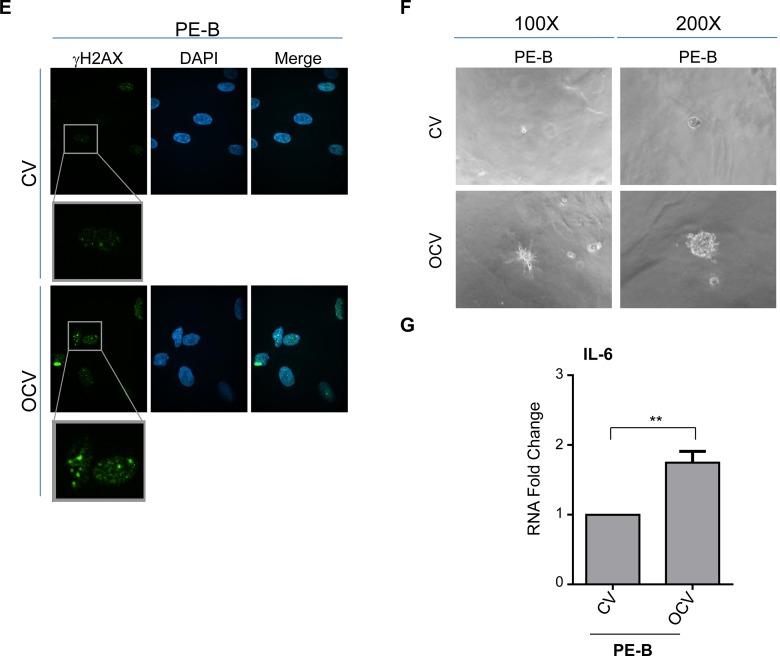

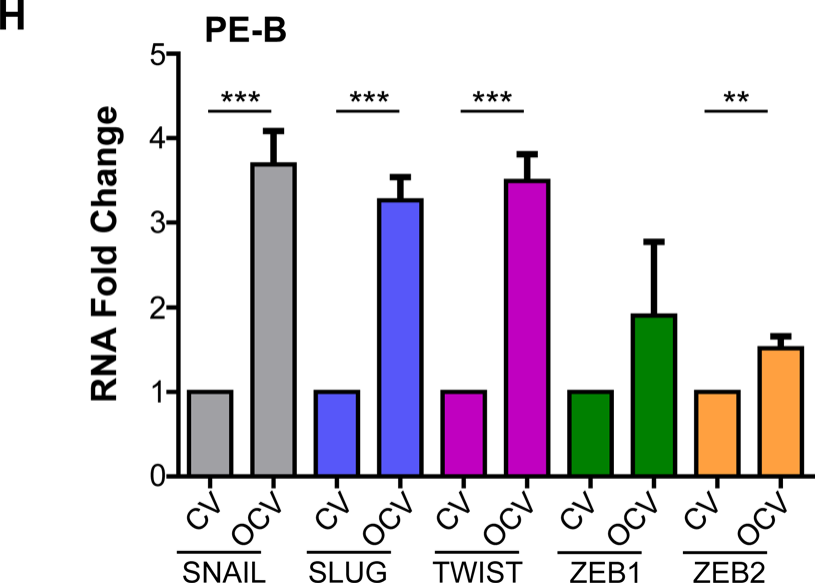
Transformation of human primary endometriotic cells (**A**) Schematic depicting the overall strategy involving retroviral infections (with control virus (CV) or oncogenic cocktail virus (OCV: comprised of HRAS^V12A^, c-MYC^T58A^, SV40 LTAg, and HA-hTERT)) to generate transformed endometriotic from primary cells (PE-A, PE-B, PE-C, and PE-D; * refers to life-extended PE-D cells with SV40 LTAg) isolated from endometriotic lesions. Two batches of transformed endometriotic cells were successfully obtained using PE-A and PE-B primary cells. The first batch of retrovirally infected cells (PE-B-CV and PE-B-OCV) were utilized to: (**B**) obtain cell lysates for western blotting with the indicated antibodies (left panel). The dotted line specifies re-run samples to avoid the possibility of detecting overlapping bands of similar molecular weights. Densitometric analyses for pAKT and pMAPK are shown in the right panels; (**C**) perform colony formation assay and images were captured following 14 days in culture (representative images are shown, three independent experiments were conducted); (**D**) perform β-galactosidase staining and images were captured at 100× magnification (representative images are shown, three independent experiments were conducted); and (**E**) assess DNA damage via γH2AX immunofluorescence staining (representative images shown were captured at 63× magnification and the images of nuclei were enlarged and cropped using PowerPoint to focus on the DNA damage foci). The second batch of retrovirally infected cells (PE-B-CV and PE-B-OCV) were utilized to: (**F**) assess the *in vitro* tumorigenic potential (by 3-dimensional morphogenesis assay in Matrigel). Representative images (from four independent experiments) were captured at 100× (left) and 200× (right) magnification; (**G**) to measure IL-6 transcript levels via real-time PCR. Three independent experiments were performed; and (**H**) assess transcript levels for genes in the EMT pathway via real-time PCR (three independent experiments were performed).

To begin to delineate the process involved in the transition from endometriotic precursors to ovarian carcinomas, long-term culturable endometriotic cells are necessary. However, due to a limitation in the availability of characterized cell lines (i.e., immortalized and/or transformed human endometriotic cells), this remains a challenge. Thus, we made attempts to generate endometriotic cell lines that could be maintained in culture long-term without reaching senescence. As described in the Methods, we obtained primary cells derived from four different human endometriotic lesions: PE-A, PE-B, PE-C and PE-D. We infected these pools of endometriotic cells with an oncogenic retrovirion cocktail (termed OCV) containing SV40 LTAg (which targets p53 and Rb), hTERT, c-MYC^T58A^, and HRAS^V12A^ (as outlined in Figure 1A) based on a similar strategy described for fimbrial epithelial cell transformation [20]. We were successful in obtaining transformants for the PE-A and PE-B endometriotic cells. We validated expression of the oncogenic markers via western blotting (Figure 1B, Supplementary Figure 1A, and 1E). While hTERT protein was undetectable (data not shown), expression of LTAg, HRAS, and c-MYC were clearly increased in OCV relative to CV infected cells. Additionally, we noted increased expression of ID1 and pAKT with reductions in pMAPK, suggestive of bypassing senescence [21]. Indeed, results from the colony formation assay (Figure 1C and Supplementary Figure 1B) and β-galactosidase staining (Figure 1D and Supplementary Figure 1C) support that CV infected cells senesced in contrast to OCV infected cells which were proliferative, lacked senescent cells, and demonstrated increased DNA damage foci upon staining with a γH2AX antibody (Figure 1E and Supplementary Figure 1D). In addition, we noted that PE-A-OCV and PE-B-OCV cells elicited *in vitro* tumorigenic potential (in the 3D morphogenesis assay after 10 days of growth) (Figure 1F and Supplementary Figure 1F). We also identified increased IL-6 mRNA in OCV infected PE-A and PE-B cells relative to controls (Figure 1G and Supplementary Figure 1G), which has been correlated with increased tumorigenicity [22]. To note, although three biological replicates were available for PE-B cells (both CV and OCV infected), statistical significance could not be determined for PE-A cells due to limitations in available numbers of CV-infected cells as a result of reaching senescence (one biological replicate). Collectively, these data indicate that we successfully obtained transformed endometriotic cells upon HRAS^V12A^ and c-MYC^T58A^ overexpression together with p53 inactivation, which are characterized by increased *in vitro* tumorigenic potential.

**Figure 2:**
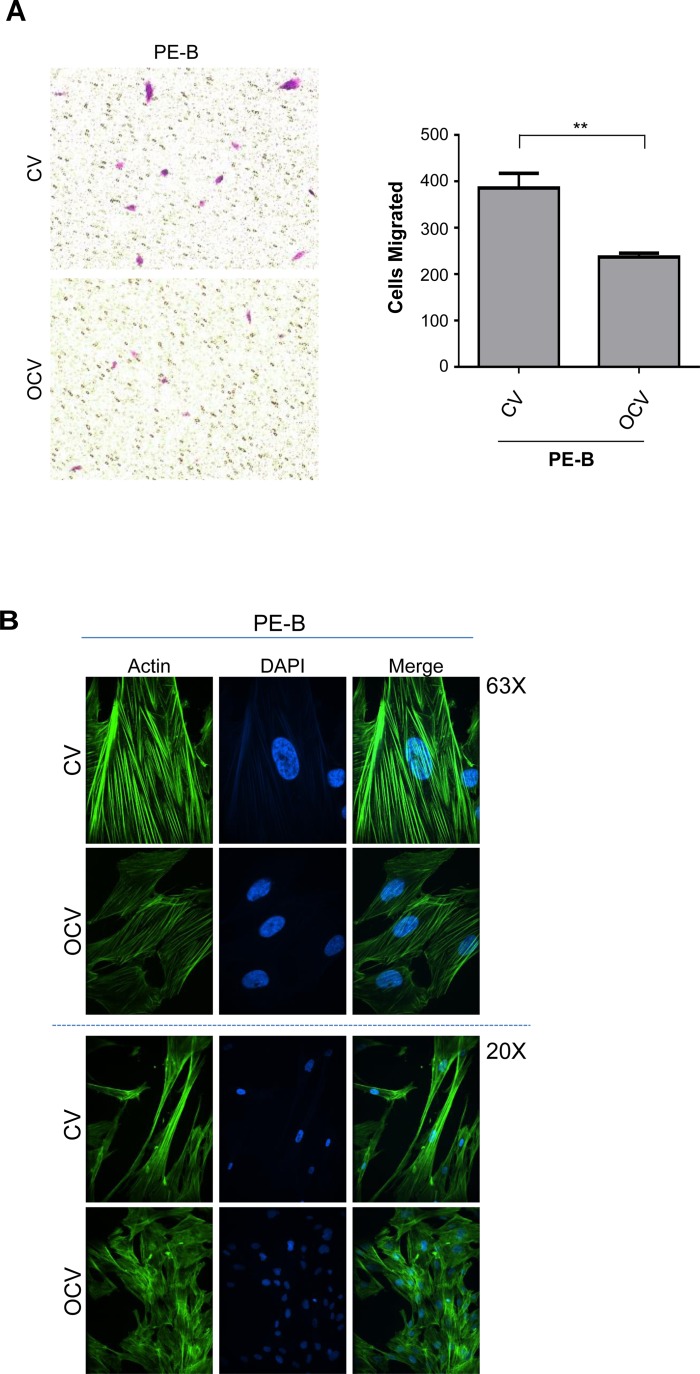

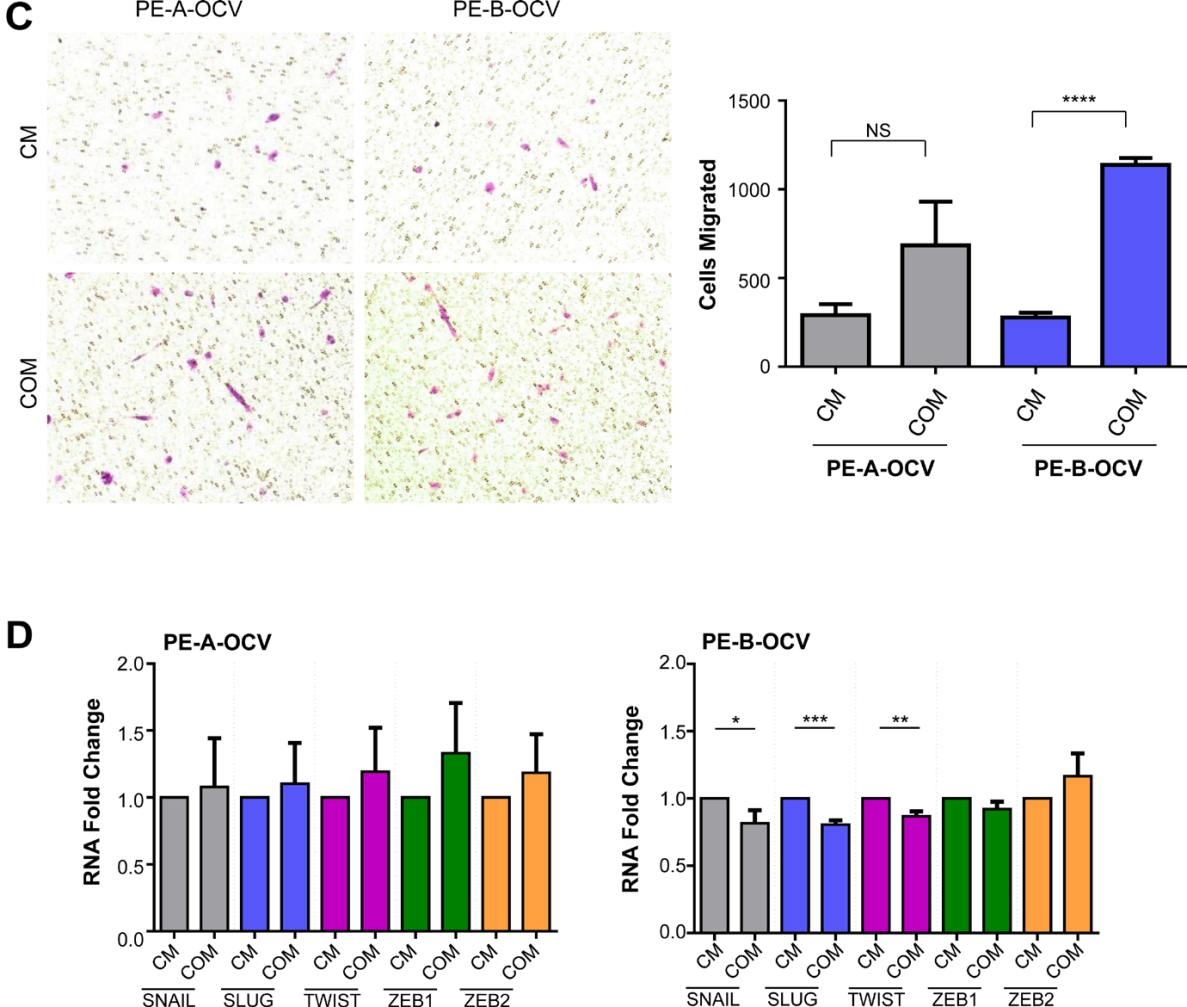

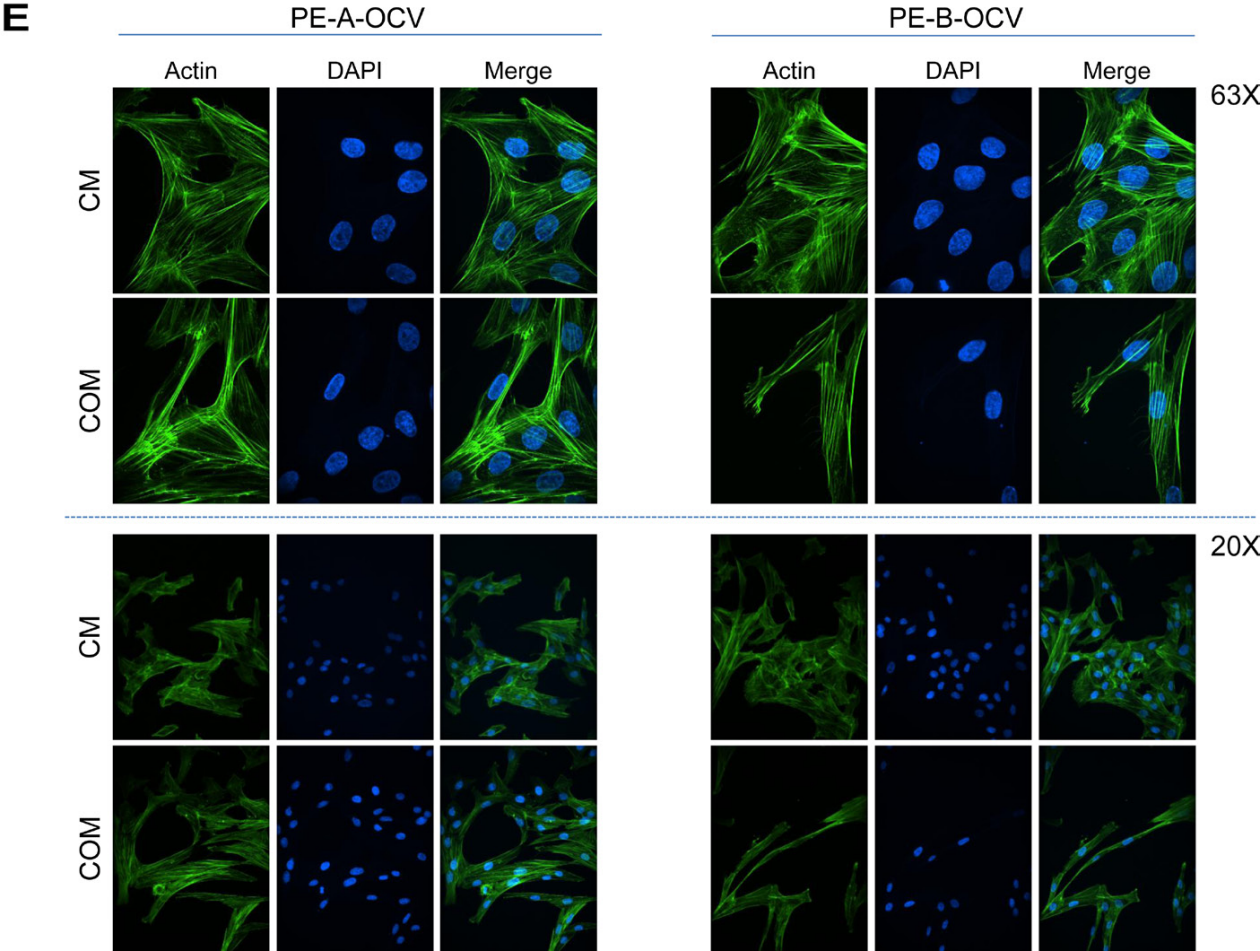
Conditioned media from senescent primary endometriotic cells promotes migration of transformed endometriotic cells The second batch of retrovirally infected cells (PE-B-CV and PE-B-OCV) were utilized to: (**A**) perform migration assay. Representative images (from four independent experiments) were captured at 100× magnification (left panel). Manual cell counts are presented in the right panel; and (**B**) assess actin filament organization using phalloidin staining. Representative images (from three independent experiments) are shown at 63× (top panel) and 20× (bottom panel) magnification. The second batch of retrovirally infected cells (PE-A-OCV and PE-B-OCV) were utilized to: (**C**) assess migration using either complete media (CM) or senescence-conditioned media (COM) as the chemoattractant. Representative images (from four independent experiments) are shown at 100× magnification (left panel). Manual cell counts are presented in the right panel; (**D**) assess transcript levels for genes in the EMT pathway via real-time PCR using RNA from these cells treated for 24 hours with CM or COM media (three independent experiments were performed); (**E**) assess actin filament organization via phalloidin staining in cells treated with CM or COM for 24 hours. Representative images (from three independent experiments) are shown at 63× (top panel) and 20× (bottom panel) magnification.

Further characterization of these transformed endometriotic cells (PE-A-OCV and PE-B-OCV) identified markedly elevated mRNA transcripts for EMT pathway genes (SNAIL, SLUG, TWIST, ZEB1, and ZEB2) (Figure 1H and Supplementary Figure 1H) relative to their CV infected counterparts suggesting that the transformed endometriotic cells may have increased migratory potential. However, we unexpectedly discovered that the OCV infected cells were less migratory (∼31–39%, *p*-value = 0.005–0.0014, for PE-A-OCV and PE-B-OCV, respectively) (Figure 2A and Supplementary Figure 2A (left and right panels)) and more cuboidal compared to CV infected cells (Figure 2B and Supplementary Figure 2B). Since senescent cells are capable of inducing cellular migration of those nearby via secretion of factors, namely the senescence-associated secretory phenotype (SASP [23]), we collected conditioned media (COM) from senescent cultures of endometriotic cells (CV-infected) and used it as a chemoattractant to assess motility (using complete media (CM) as a control). As shown in Figure 2C (left and right panels), we noted increased migratory capacity of transformed endometriotic cells with COM media (PE-A-OCV (2.4-fold, *p* = 0.0550) and PE-B-OCV cells (4.1-fold *p* < 0.0001)) compared to CM. This increased migratory phenotype in response to COM media was not accompanied by dramatic alterations in EMT marker mRNA expression in the PE-A-OCV and PE-B-OCV cells relative to CM-treated (Figure 2D). We next investigated whether the above observed phenomena were accompanied by changes in cellular morphology via staining with phalloidin; indeed, COM mediated an elongated cell morphological change in the transformed endometriotic cells compared to CM-treated cells (Figure 2E). Collectively, these data suggest that the senescent endometriotic cells are capable of increasing the migratory capacity of nearby cells.

### Transformed endometriotic cells elicit alterations in iron pathway markers including NCOA4

Oncogenic alterations (associated with increased growth potential) has been reported to lead to changes in intracellular iron levels as a result of changes in the expression of iron pathway signaling mediators such as the transferrin receptor (CD71), ferroportin (FPN), and ferritin complex amongst several others [24–27]. However, whether oncogenic transformation of endometriotic cells (as a precursor lesion for specific ovarian cancer subtypes) leads to changes in the expression of iron pathway signaling mediators including NCOA4, a novel intracellular regulator of intracellular iron levels [28, 29], has not yet been investigated. As described earlier, there exists two well-studied NCOA4 isoforms (NCOA4α and NCOA4β) with opposing functions in breast and prostate cancers [17, 18]. The alignment of NCOA4β (missing an internal 327 amino acid sequence) with a published NCOA4α protein sequence (accession #: Q13772) is presented in Supplementary Figure 3A. The role of these isoforms in ovarian cancer remains uninvestigated other than one report of increased NCOA4 mRNA levels in ovarian tumor specimens relative to ovarian surface epithelium (via *in situ* hybridization [19]).

**Figure 3:**
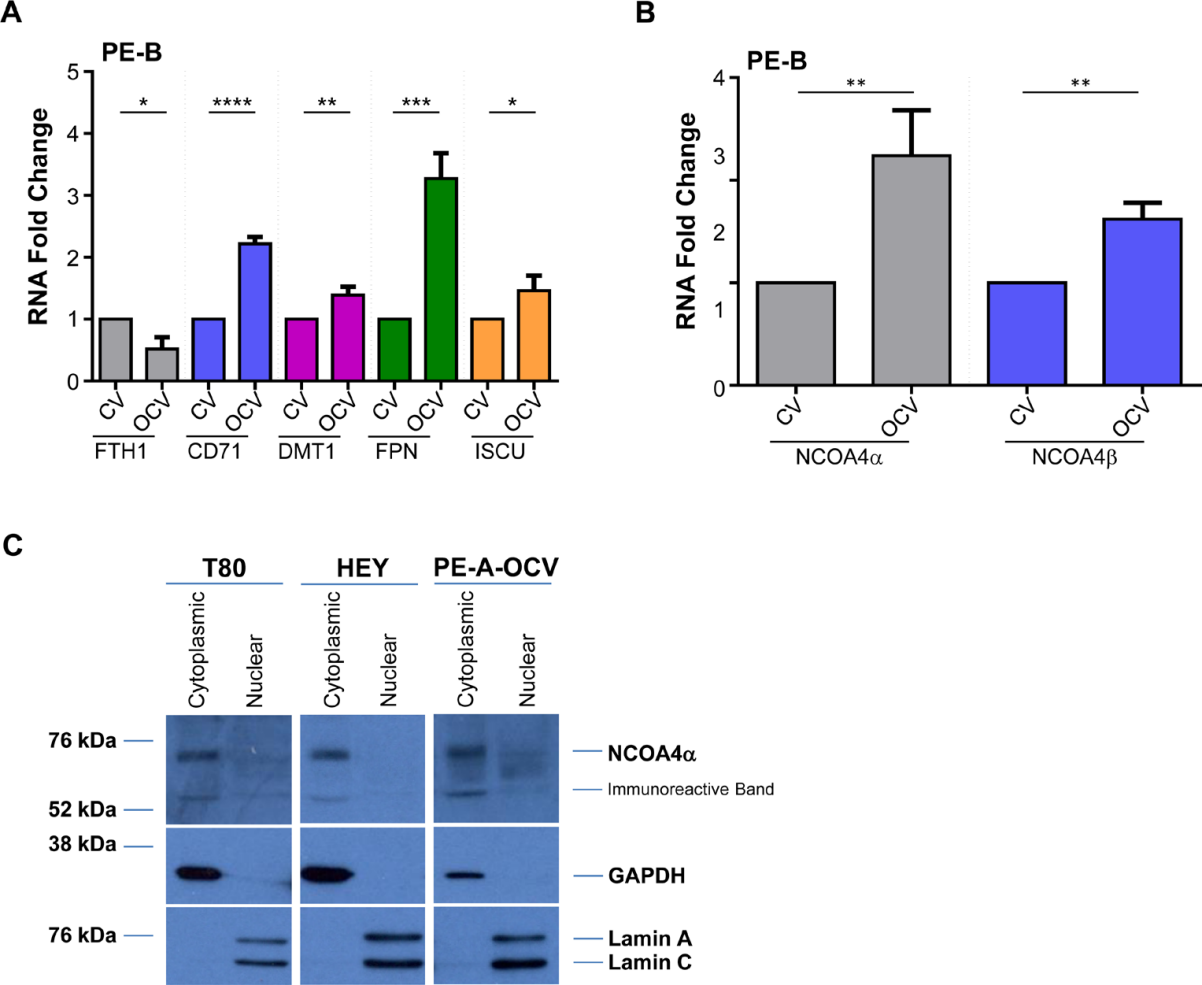

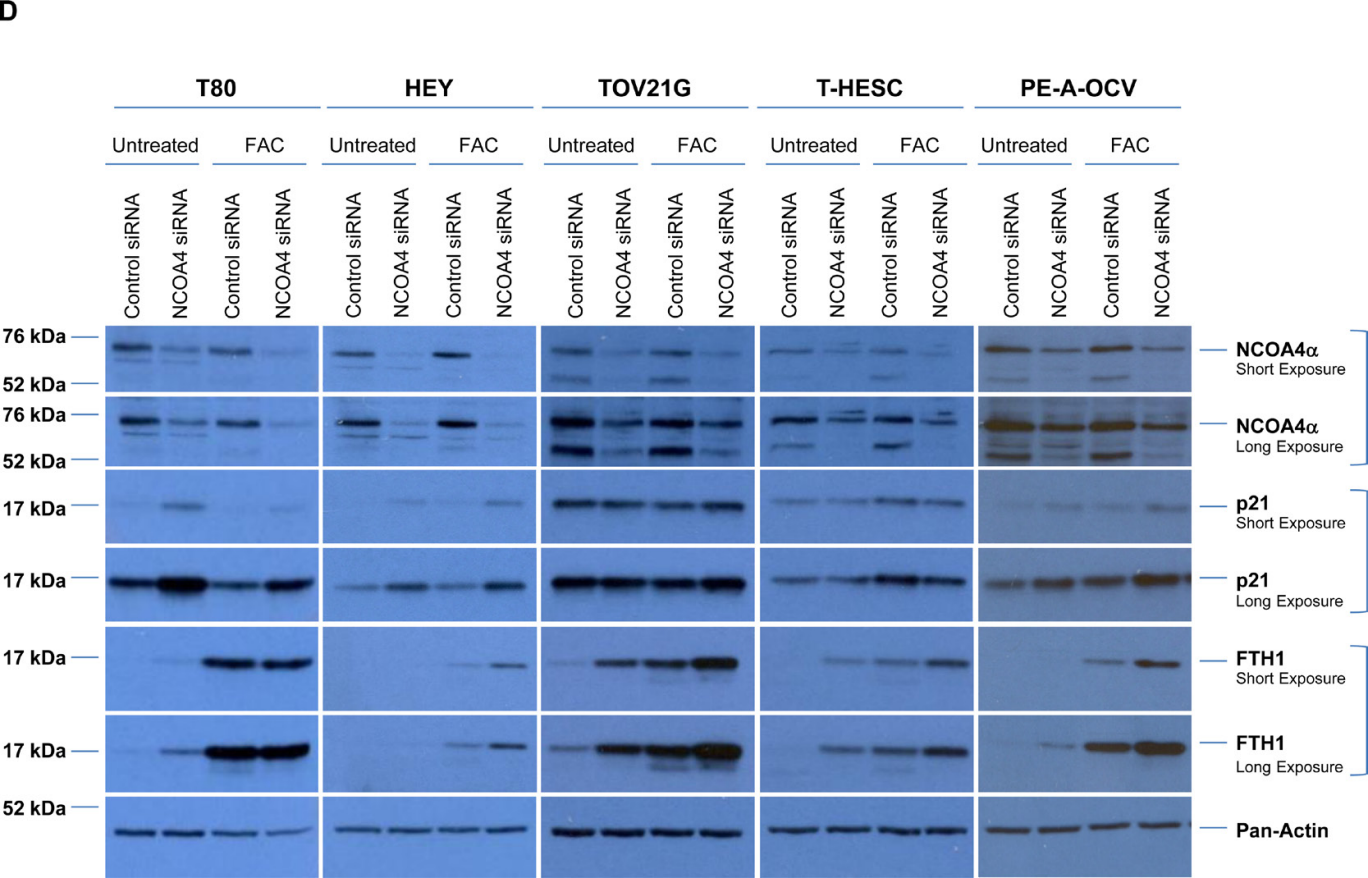

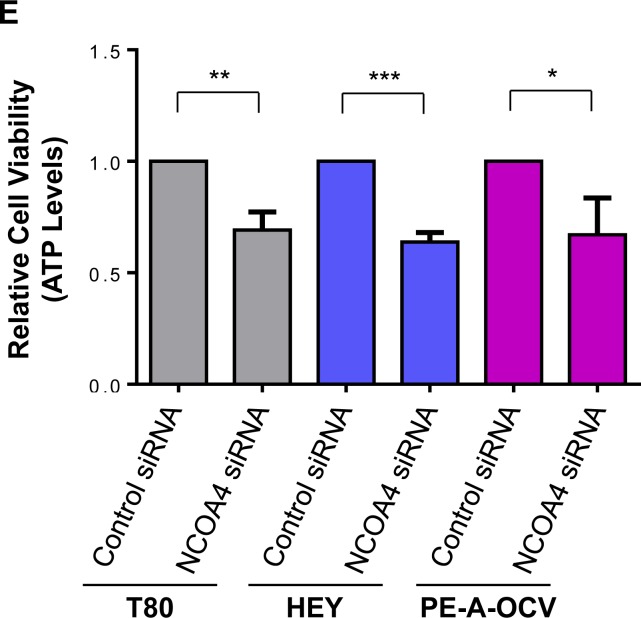

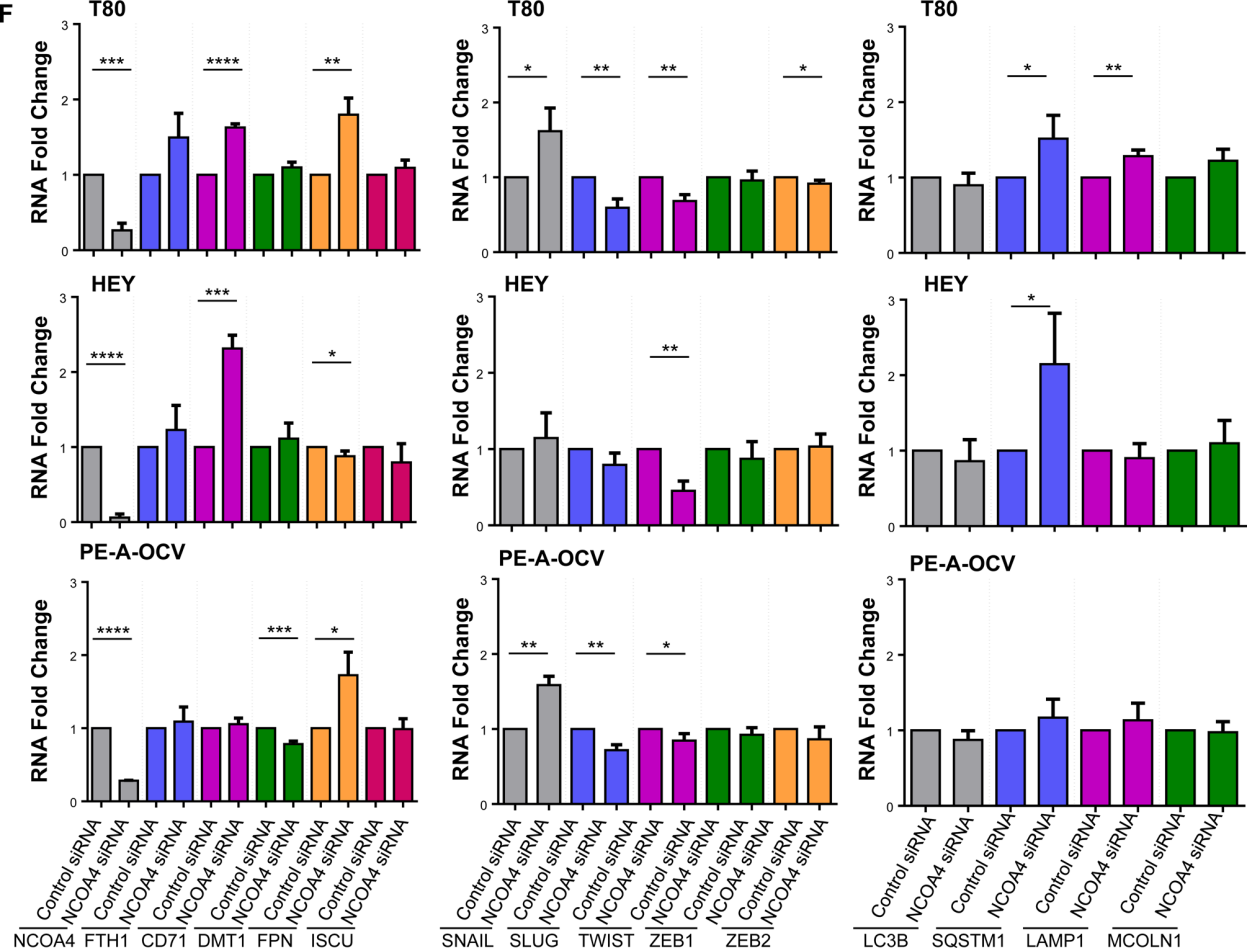
NCOA4 knockdown reduces cellular viability in T80, HEY, and PE-A-OCV cells Transcript levels for (**A**) genes in the iron pathway including (**B**) NCOA4α and NCOA4β were assessed via real-time PCR using RNA extracted from the second batch of PE-B-CV and PE-B-OCV cells. Three independent experiments were performed. (**C**) Western analyses of cytoplasmic and nuclear fractions from T80, HEY, and the first batch of PE-A-OCV cells were performed using the indicated antibodies. Data shown is representative of three independent experiments. (**D**) Cell lysates from NCOA4 siRNA treated T80, HEY, TOV21G, T-HESC, and the second batch of PE-A-OCV (treated for 18 hours with 250 μM FAC) were analyzed by western blotting using the indicated antibodies. Data shown are representative of three independent experiments. (**E**) Cellular viability (via ATP measurements) was assessed upon NCOA4 knockdown in T80, HEY, and the second batch of PE-A-OCV (2nd batch) cells. Three independent experiments were performed. (**F**) Using RNA isolated from T80, HEY and the second batch of PE-A-OCV treated with NCOA4 siRNA, transcript levels for genes in the iron, EMT, lysosome/autophagy pathways were measured via real-time PCR. Three independent experiments were performed.

In our transformed endometriotic cells, we observed multiple alterations in iron pathway markers including an increase in CD71 and DMT1 (PE-A-OCV and PE-B-OCV) as well as FPN and ISCU (PE-B-OCV), with a reduction in FTH1 mRNA (PE-A-OCV and PE-B-OCV) (Figure 3A and Supplementary Figure 3B). To investigate the expression of the two NCOA4 isoforms in these cells, we first needed to generate custom-designed probes and primers to target NCOA4α (spanning the junction between exon 8 and exon 9) and NCOA4β (spanning the junction between exon 7 and exon 9 (Supplementary Figure 3C depicts the binding location of these probes)) for real-time PCR. The specificity of these probes was assessed using RNA isolated from NCOA4α overexpressing T80 cells and NCOA4β overexpressing HEY cells. As shown in Supplementary Figure 3D, these probes could distinguish between these two splice variants (NCOA4α and NCOA4β mRNA mRNA (∼8-fold and ∼147-fold increase, respectively) relative to their corresponding controls. As shown in Supplementary Figure 3E and 3F, a corresponding ∼2-fold and ∼6-fold increase in NCOA4α (70 kDa) and NCOA4β (35 kDa) protein was noted. We next identified a ∼2.2–2.6-fold (*p* = 0.0084) increase for NCOA4α and a ∼1.3–1.6-fold increase (*p* = 0.0026) for NCOA4β in PE-A-OCV and PE-B-OCV relative to non-transformed cells (Figure 3B and Supplementary Figure 3G). These data indicate that cellular transformation of primary endometriotic cells leads to increases in the mRNA levels of both NCOA4 isoforms.

### NCOA4 is localized predominantly to the cytoplasm and modulates cellular viability of T80, HEY, and PE-A-OCV cells

Since there is evidence describing the localization of NCOA4 isoforms in the cytosolic and nuclear compartments of prostate tissues (possibly to mediate its function as a hormone receptor coactivator [16, 30] [31, 32]), we next investigated the localization of this iron mediator in our gynecological cells. As shown in Figure 3C, we identified that NCOA4α was localized predominantly to the cytoplasmic fraction (with no detectable expression in the nuclear fraction) in T80, HEY, and PE-A-OCV cells; to note, NCOA4β protein was not detected in these cells (data not shown).

To investigate the role of NCOA4 in gynecological cell survival and determine whether its activity is altered upon exposure to iron, we reduced NCOA4 levels in normal/benign immortalized cells (T80 and T-HESC), in transformed human endometriotic cells (PE-A-OCV), and in OVCA cell lines (HEY and TOV21G) using an siRNA approach. In addition, we tested the effect of NCOA4 knockdown in the absence or presence of ferric ammonium citrate (FAC) as we previously tested the effects of iron overload conditions (using FAC) in HEY cells [33]. As shown in Figure 3D, NCOA4 protein was reduced upon knockdown by ∼65% in T80, ∼75% reduction in HEY, ∼55% in T-HESC, ∼65% in TOV21G, and ∼50% in PE-A-OCV (we identified reduced NCOA4 mRNA using a real-time PCR probe/primer detecting all isoforms (∼74% in T80, ∼94% in HEY, and ∼71% in PE-A-OCV cells (Figure 3F); see Supplementary Figure 3H for NCOA4 isoform specific probes/primers real-time PCR data)). NCOA4 protein was not further altered by FAC (Figure 3D). The NCOA4 antibody also detected a ∼55 kDa immunoreactive band that has been previously reported [34] which we found to be reduced upon NCOA4 knockdown; the identity of this band remains unclear. Upon reduction of NCOA4, we observed increases in FTH1 protein in all cell lines whereas increases in p21 (cyclin-dependent kinase inhibitor which is elevated under conditions of growth arrest [35]) were identified in T80 (∼3.5-fold), HEY (∼2-fold), and PE-A-OCV cells (∼2-fold). In this respect, we determined that there was an association between elevated p21 (following NCOA4 knockdown) with inactivated p53 (in T80 and PE-A-OCV cells which express LTAg which binds to p53 (results not shown and [36])) or undetectable p53 (in HEY cells (results not shown)) whereas T-HESC and TOV21G have wild type p53 levels (results not shown and [37]) and were not associated with changes in p21 levels with reduced NCOA4. Knockdown of NCOA4 reduced cellular viability by ∼31%, 36%, and 33% in T80, HEY, and PE-A-OCV cells, respectively (Figure 3E).

We additionally observed increases in FTH1 (T80 cells), CD71 (T80 and HEY cells), and FPN (T80 and PE-A-OCV cells) mRNA levels (Figure 3F). Since NCOA4 is a regulator of ferritinophagy, we also examined the transcript profile for genes involved in autophagosome or lysosome formation. In this regard, we identified increases in SQSTM1 (∼1.5-fold (T80) and ∼2-fold (HEY)) involved in autophagic flux [38] and in MCOLN1 (∼1.2-fold (T80)) involved in iron transport to lysosomes [39]. Since cellular viability was reduced with NCOA4 siRNA, we next investigated the effect of NCOA4 knockdown on markers of tumorigenicity including those involved in EMT progression and identified an increase in SNAIL (∼1.6-fold in both T80 and PE-A-OCV cells) with a decrease in SLUG (∼28% in PE-A-OCV cells) and TWIST (∼55% in HEY cells) transcripts which further implicate NCOA4 in promoting cellular survival.

### Expression of NCOA4 is regulated by factors present within serum

**Figure 4:**
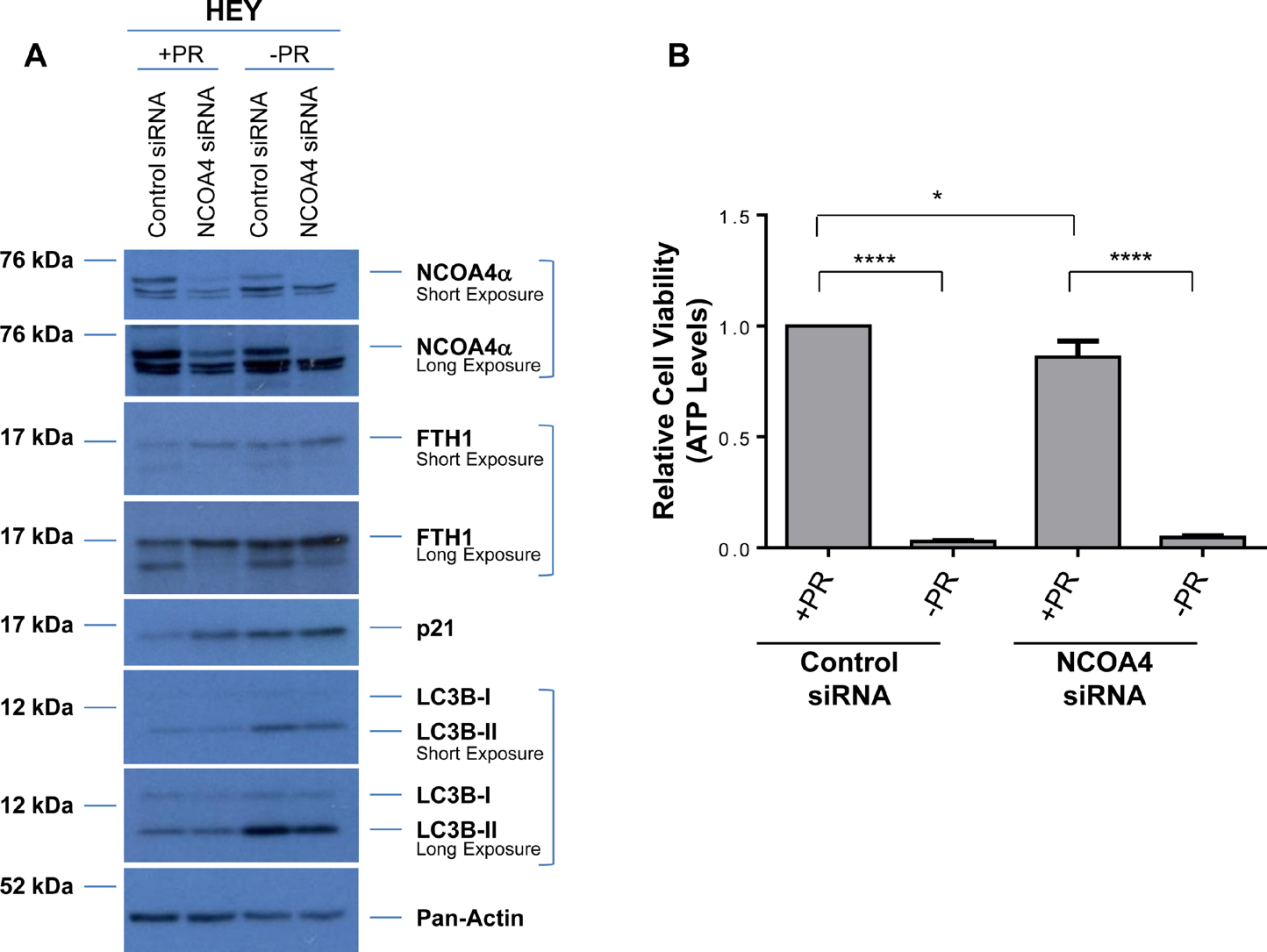

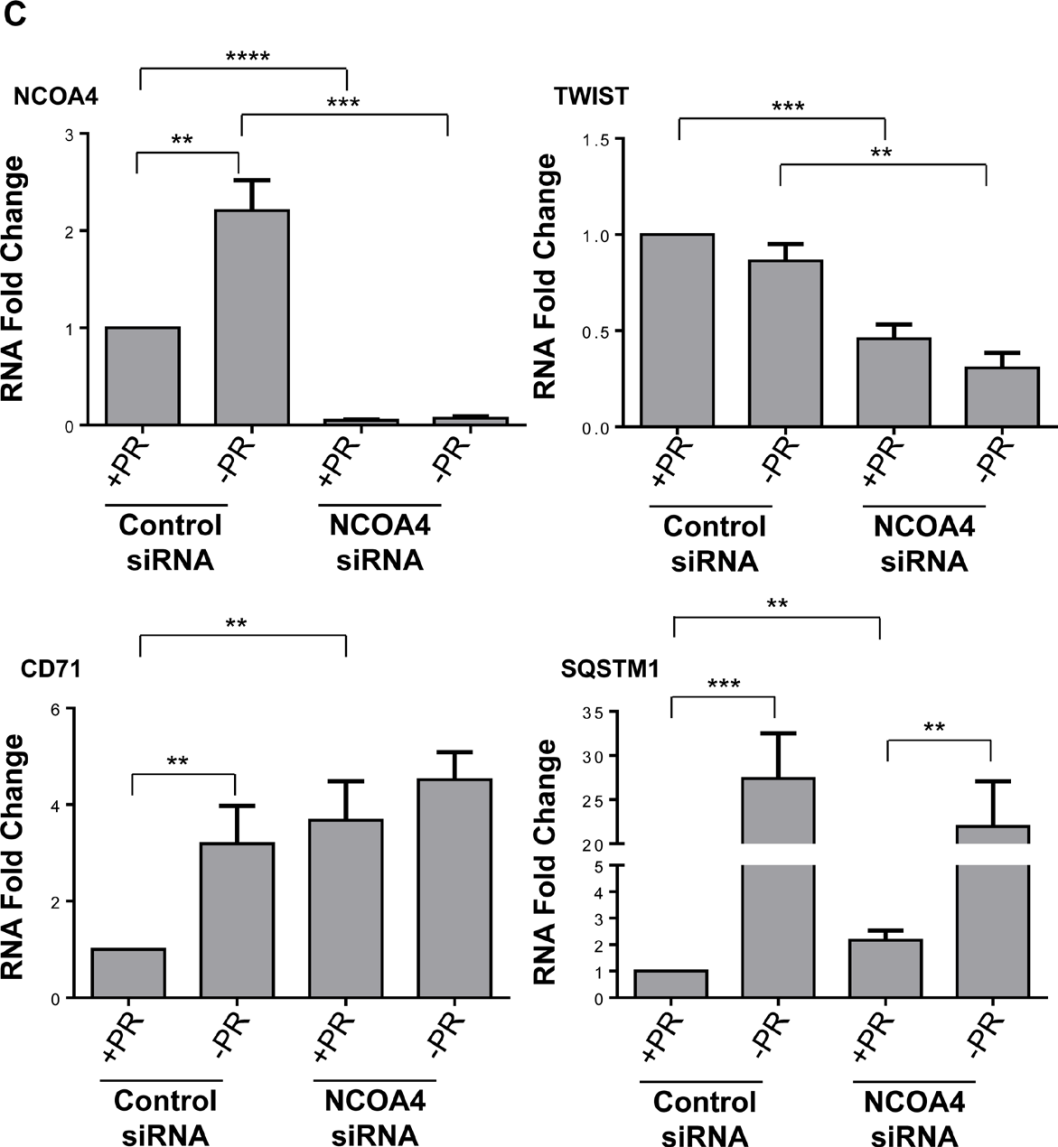
Cellular responses to NCOA4 knockdown is altered in response to factors present in FBS (**A**) HEY cells were treated with control or NCOA4 siRNA in the absence (-PR) or presence (+PR) of serum factor-replete media. Cell lysates were analyzed by western blotting using the indicated antibodies. Data shown is representative of three independent experiments. (**B**) Cellular viability (assessed via ATP levels) of HEY cells was assessed following knockdown of NCOA4 (via siRNA) and treatment for 24 hours with +PR or -PR media. Three independent experiments were performed. (**C**) RNA was isolated from HEY cells treated with NCOA4 siRNA in +PR or -PR media for 24 hours and utilized for real-time PCR to assess transcript levels for total NCOA4, CD71, TWIST, and SQSTM1. Three independent experiments were performed. (**D**) Cytoplasmic and nuclear fractions from HEY cells treated for 24 hours with +PR or −PR media were analyzed by western blotting with the indicated antibodies. Data shown is representative of three independent experiments.

Fetal bovine serum, commonly used for promoting cell growth and attachment, consists of multiple factors (such as growth factors, cytokines, hormones, and other lipophilic proteins) which can be removed by charcoal-dextran treatment [40]. To assess whether our observed biochemical and functional changes mediated by NCOA4 are dependent on these above-described factors, we utilized media deficient in phenol red and charcoal-dextran treated FBS (-PR) following NCOA4 knockdown. We completed these analyses using HEY as these cells showed a more robust cellular effect in response to NCOA4 knockdown relative to T80 and PE-A-OCV cells. As shown in Figure 4A, the use of –PR media increased LC3B-II, FTH1, and p21 basal protein expression relative to complete (+PR) media; FTH1 and p21 protein were not further altered upon NCOA4 knockdown in –PR media. We also noted that cell viability was reduced in HEY cells in –PR relative to +PR conditions, with either control or NCOA4 siRNA (Figure 4B). While we observed significant reduction of NCOA4 mRNA upon NCOA4 knockdown under both media conditions (+PR and –PR), the NCOA4 mRNA (assessed with the commercially available Taqman probe and primers) increased by ∼2-fold (*p* = 0.0027) in –PR conditions suggesting that the presence of these serum factors could potentially inhibit NCOA4 transcription (Figure 4C, top left panel). Although CD71 mRNA increased upon NCOA4 knockdown (relative to control siRNA) in +PR media (∼3.6-fold, *p* = 0.0046), this increase was reduced in -PR media (∼1.4-fold, *p* = 0.0761). We noted a further reduction in TWIST mRNA in –PR media upon NCOA4 siRNA treatment relative to control siRNA (∼65% reduction, *p* = 0.0012 compared to ∼55% reduction, *p* = 0.0002). Further, SQSTM1 mRNA was not as markedly increased with NCOA4 siRNA (compared to control siRNA) in -PR media (∼10-fold (*p* = 0.0027) relative to ∼27-fold (*p* = 0.0009)). Collectively, these results implicate the involvement of these factors in the functional responses of NCOA4 in ovarian cancer cells.

### Overexpression of NCOA4β reduces colony forming potential

**Figure 5:**
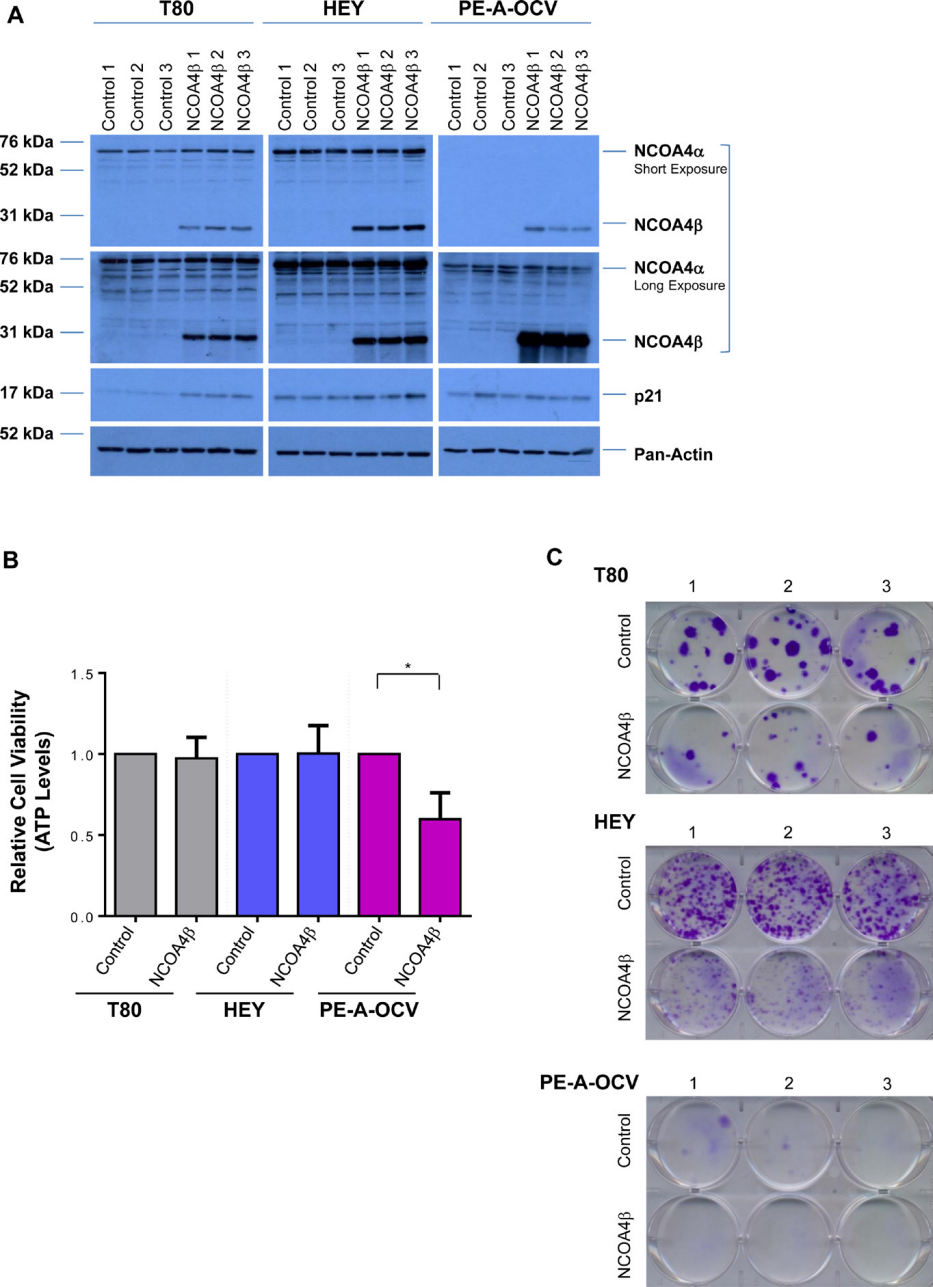

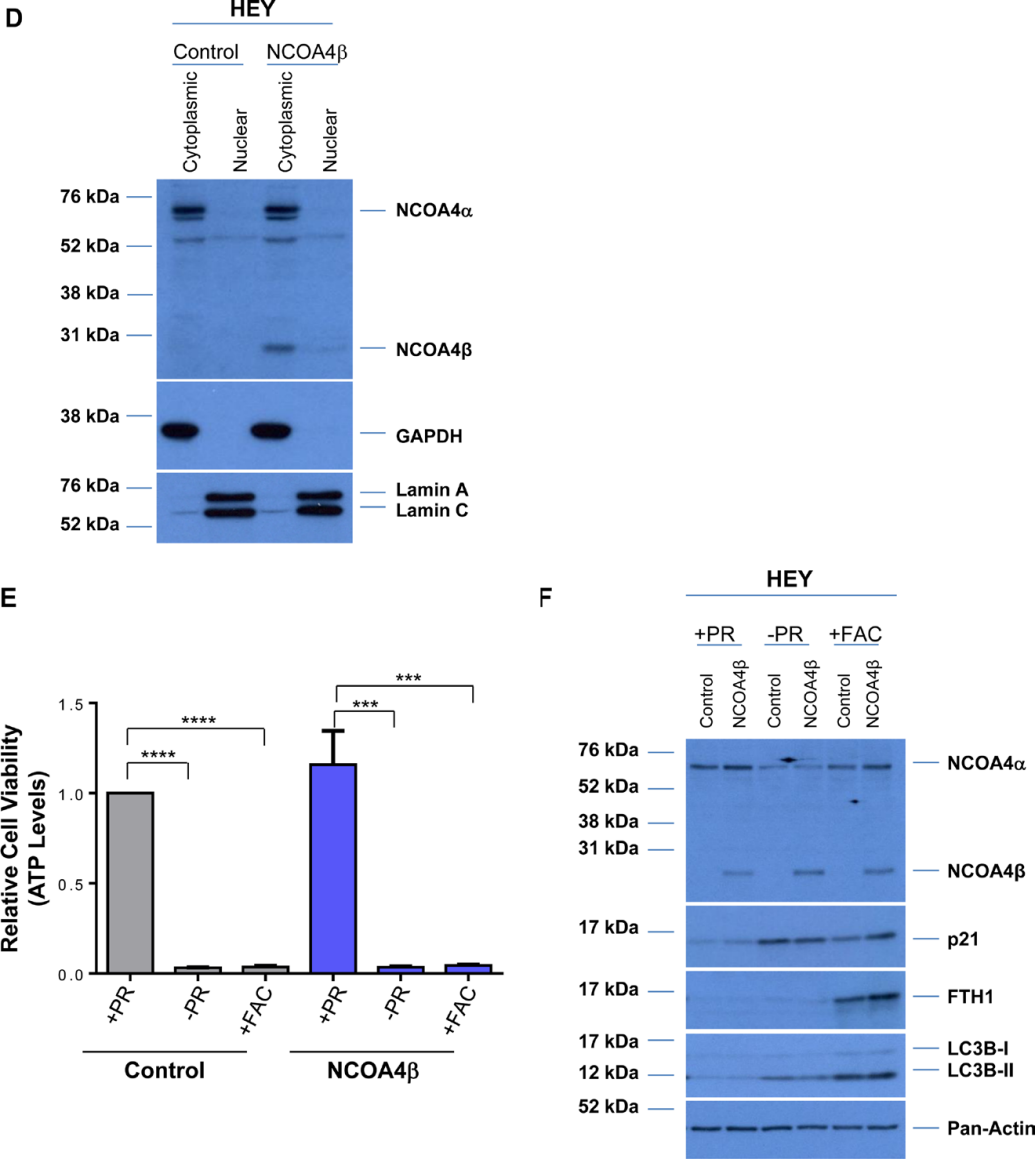

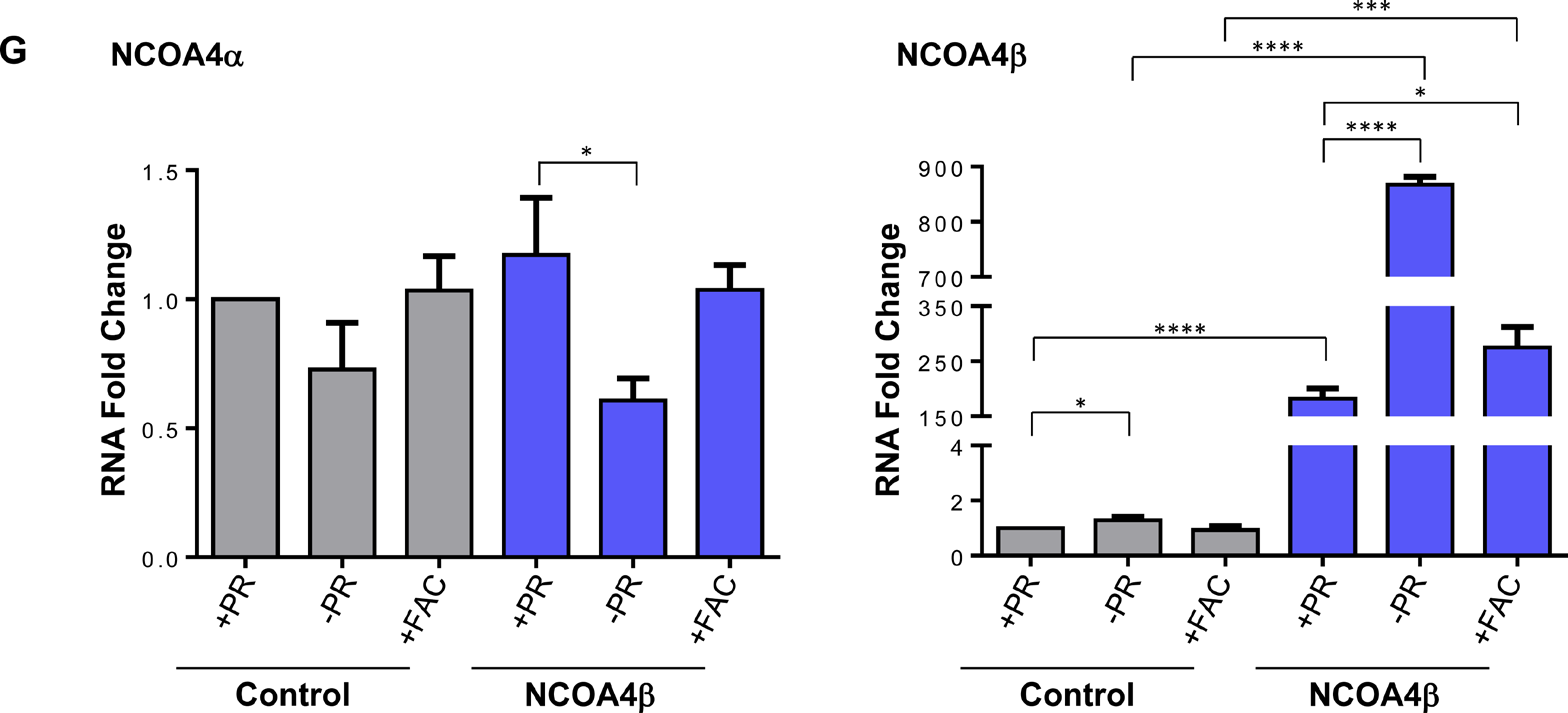
NCOA4β overexpression in T80, HEY, and PE-A-OCV cells reduces colony forming ability Control and NCOA4β overexpressing T80, HEY, and the first batch of PE-A-OCV cells were utilized to: (**A**) analyze protein lysates by western blotting with the indicated antibodies. Three independent replicates are shown; (**B**) analyze cytoplasmic and nuclear fractions by western blotting with the indicated antibodies. Data shown is representative of three independent experiments; (**C**) assess cellular viability (measured by ATP levels). Three independent experiments were performed; and (**D**) to assess colony formation ability after 14 days in culture. Representative images (from three independent experiments) are shown. Using control and NCOA4β overexpressing HEY cells: (**E**) cellular viability (measured via ATP levels) was assessed following a 3 day exposure to –PR media or 250 μM FAC. Three independent experiments were performed; (**F**) cell lysates (in cells treated with serum factor-depleted (-PR) media for 24 hours or 250 μM FAC for 18 hours) were analyzed by western blotting with the indicated antibodies. Data shown is representative of three independent experiments; and (**G**) Transcript levels for NCOA4α and NCOA4β were assessed via real-time PCR (cells exposed to -PR media for 24 hours or 250 μM FAC for 18 hours). Three independent experiments were performed.

Since NCOA4α appears to regulate cellular viability in our gynecological cells (see Figure 3D and 3E), we next investigated whether NCOA4β could elicit antagonistic function similar to that reported in breast and prostate cancers [17, 18]. We thus generated NCOA4β overexpressing T80, HEY, and PE-A-OCV cell lines. There were only slight changes in p21 protein expression (in T80 and HEY; Figure 5A), and no changes in short-term viability with the exception of PE-A-OC cells which elicited a ∼40% reduction in growth (*p* = 0.0129) (Figure 5B). However, we did note a marked reduction in colony forming ability (Figure 5C). NCOA4β was primarily cytoplasmic in localization (Figure 5D) similar to prostate specimens [17, 31]. NCOA4β overexpression did not alter cellular response of HEY cells to charcoal-dextran treated FBS containing media (-PR [41]) and iron overload conditions (250 μM FAC [33, 42]) (Figure 5E). However, we did observe that p21 protein was increased in –PR media (independently of NCOA4β overexpression) and in FAC-treated NCOA4β overexpressing cells (Figure 5F). FAC treatment increased FTH1 protein (as expected) and was independent of NCOA4β overexpression. In contrast, LC3B-II was increased in response to -PR conditions and in response to FAC but was reduced with NCOA4β overexpression in –PR conditions (Figure 5F). We additionally observed that NCOA4α protein was reduced and NCOA4β protein was increased -PR relative to +PR replete media (Figure 5F). Specifically, in control cells relative to +PR, -PR media resulted in a reduction in NCOA4α (∼27%, *p* = 0.0600) and an increase in NCOA4β mRNA (∼1.3-fold, *p* = 0.0191) levels (Figure 5G). Further, in NCOA4β overexpressing cells relative to +PR, –PR conditions resulted in a reduction in NCOA4α (∼48%, *p* = 0.0145) and an increase in NCOA4β mRNA (∼5-fold, *p* < 0.0001) levels. We noted that these mRNA changes were consistent at the protein level (see Figure 5F). These results suggest that NCOA4β is negatively regulated by the presence of factors present in serum.

### NCOA4 protein expression is increased in malignant ovarian cancer cell lines and in subtypes of human OVCA

**Figure 6:**
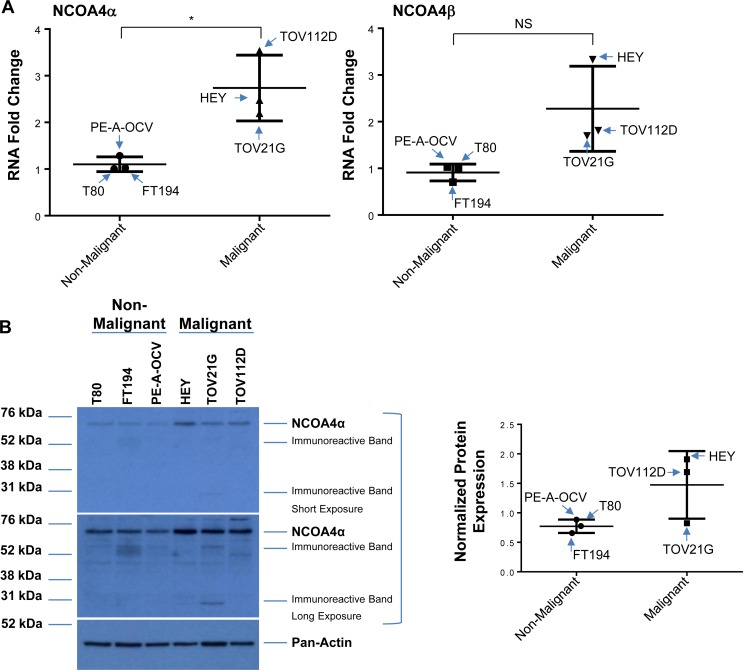

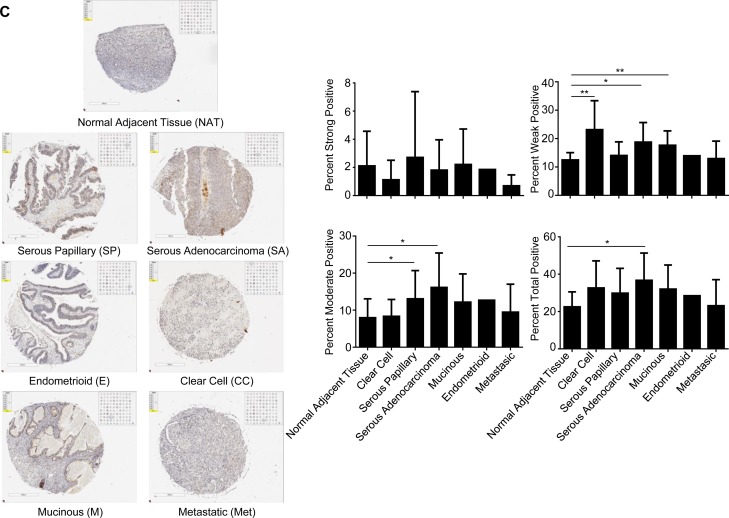
Increased NCOA4 expression in malignant gynecological cells and human OVCA tissues (**A**) RNA isolated from T80, FT194, and PE-A-OCV (non-malignant) and HEY, TOV21G, and TOV112D (malignant) cells were used to assess transcript levels for NCOA4α and NCOA4β via real-time PCR. Three independent experiments were performed. (**B**) Cell lysates from these non-malignant and malignant cell lines were analyzed by western blotting using the indicated antibodies. Densitometric analyses is shown in the right panel. Data shown is representative of three independent experiments. (**C**) Protein expression of NCOA4 was assessed in a human ovarian cancer TMA. Positive analyses was completed using the scanned image in ImageScope. Representative images of individual cores were captured at 6× magnification (left panel) and positive core analyses is presented in the right panel.

To the best of our knowledge, there is limited information on the expression of NCOA4 in clinical specimens; one report describes an increase in NCOA4 mRNA in invasive ovarian carcinomas (compared to non-malignant controls) via *in situ* hybridization [19]. We therefore assessed the expression of NCOA4 splice variants in patient-derived cell lines and ovarian cancer tissue specimens. With regard to cell lines, we assessed non-malignant (T80, FT194, and PE-A-OCV) and malignant (HEY, TOV21G, and TOV112D) gynecological cell types in which we identified increased NCOA4α (∼2.5-fold, *p* = 0.0173) and NCOA4β (∼2.5-fold, *p* = 0.06) mRNA in malignant compared to non-malignant cell lines (Figure 6A). We also identified increased NCOA4α protein in malignant gynecological cells (∼2.5- and ∼2.9-fold in HEY (relative to T80 and FT194, respectively, as precursors, and ∼1.9-fold in TOV112D (relative to PE-A-OCV as precursor)) (Figure 6B). As shown in Figure 6C, immunohistochemical staining for NCOA4 revealed that it was significantly increased in a subset of ovarian tumors relative to normal adjacent tissues: 1) serous adenocarcinoma (total, moderate, and weak positive), 2) serous papillary (moderate and weak positive), and 3) mucinous (weak positive). Overall, these data show that NCOA4 mRNA (NCOA4α and NCOA4β) as well as protein (NCOA4α) expression is increased in ovarian cancers relative to controls.

### NCOA4 protein is regulated via a proteasome- and autophagy-independent mechanism

**Figure 7:**
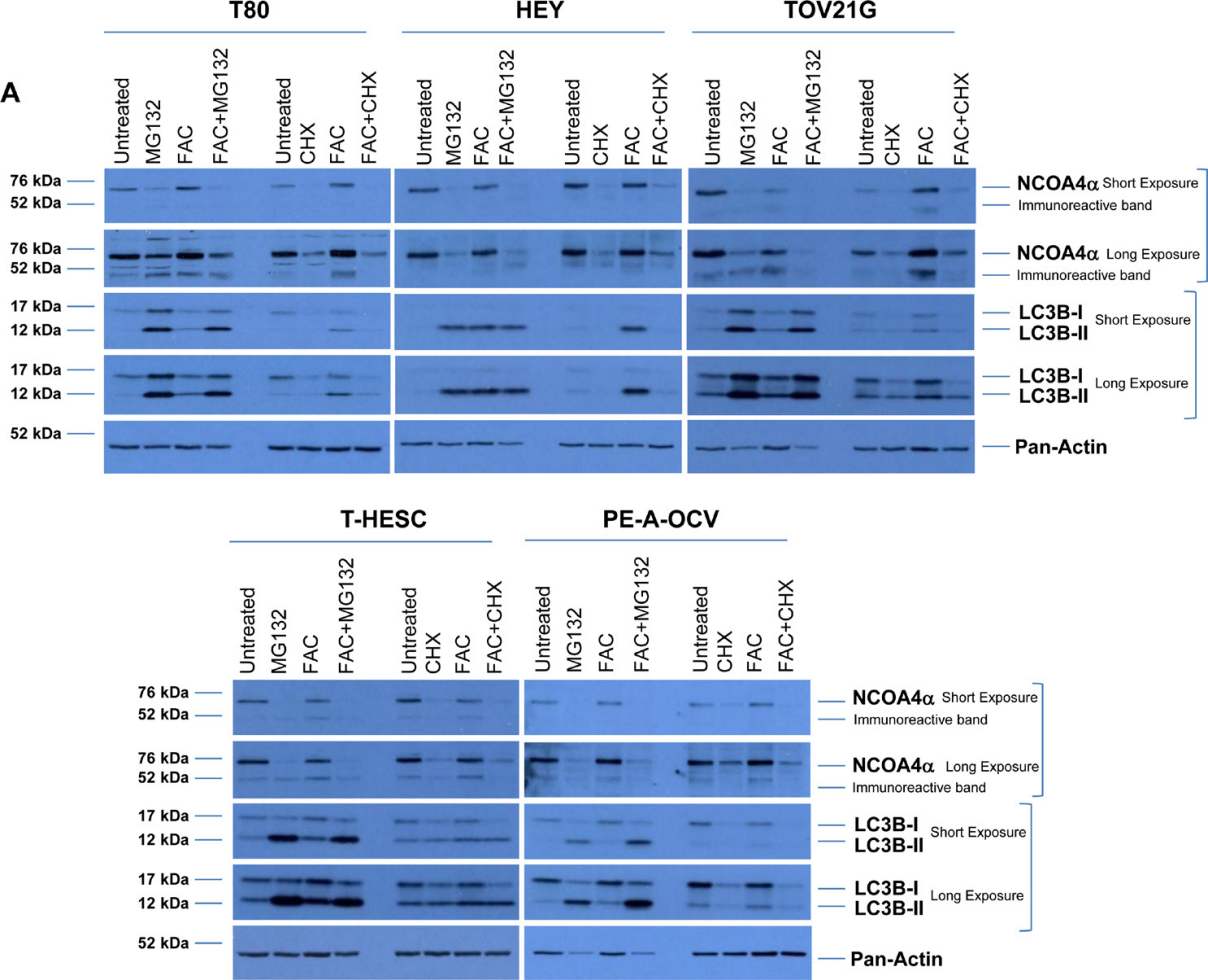

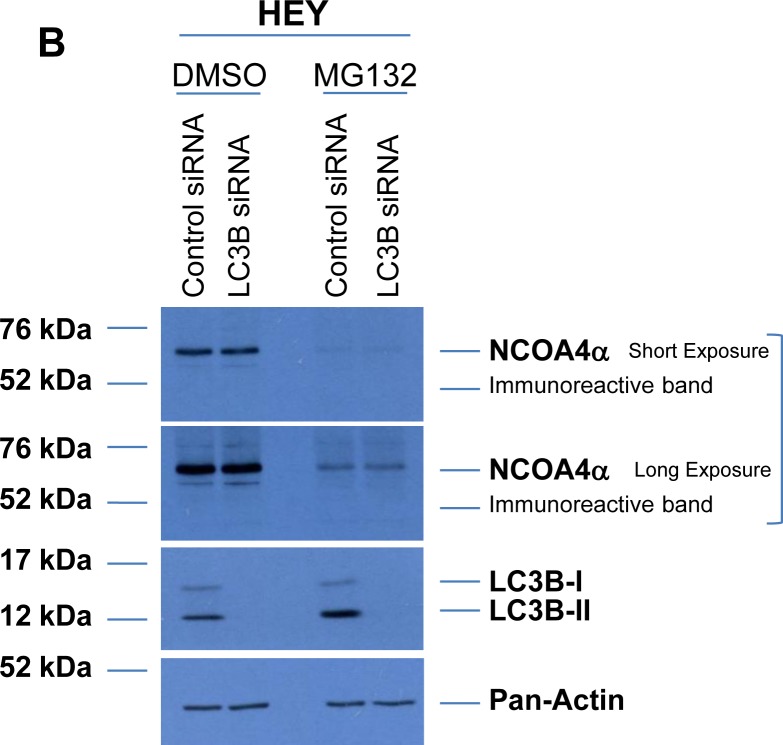

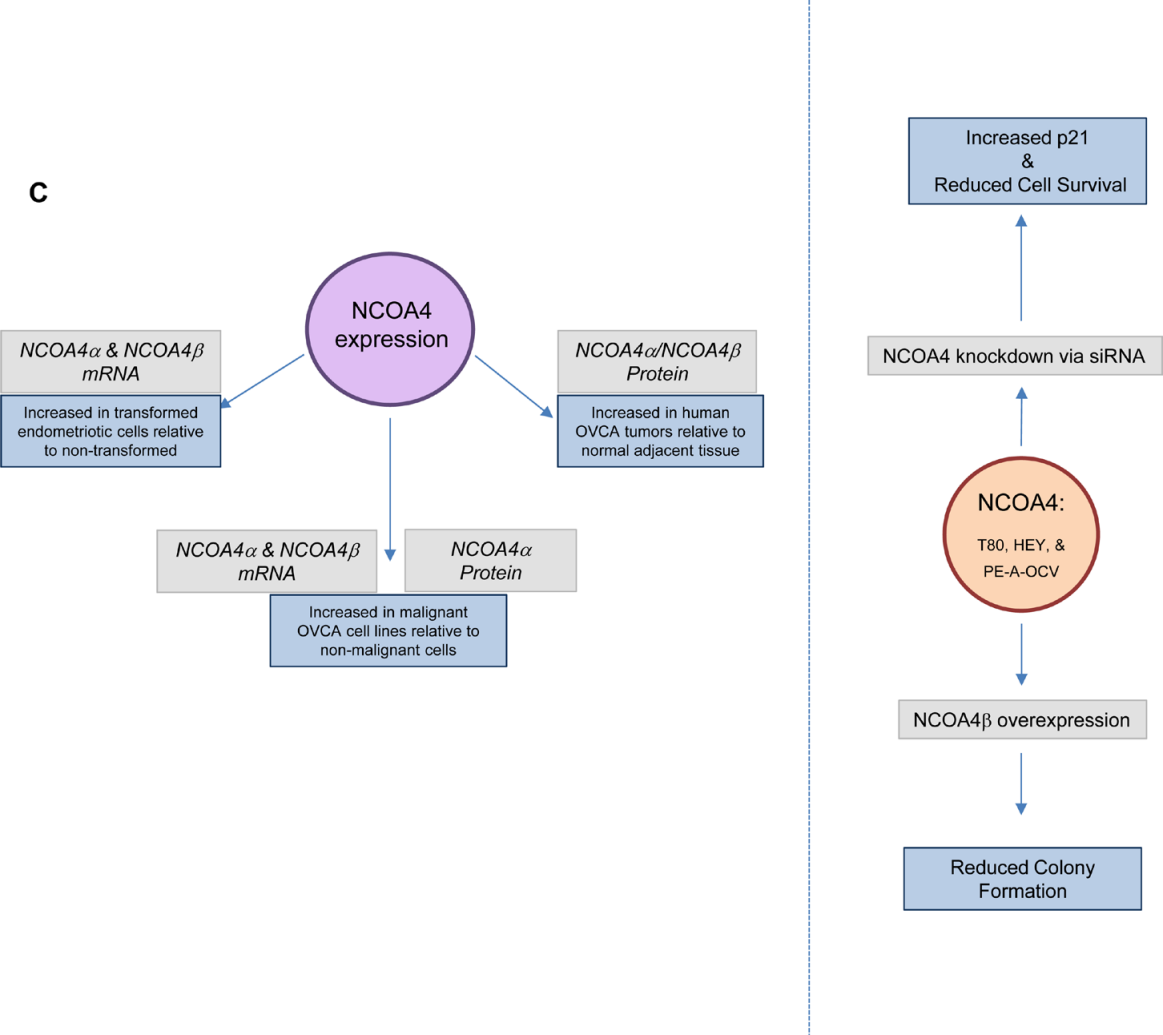
NCOA4 protein is regulated in a proteasome- and autophagy-independent manner (**A**) Cell lysates from untreated T80, HEY, TOV21G, T-HESC, and PE-A-OCV or cells treated for 18 hours with 5 μM MG132, 2 μg/ml CHX, 250 μM FAC or a combination of MG132 with FAC or CHX with FAC were analyzed by western blotting with the indicated antibodies. Data shown is representative across three independent experiments. (**B**) Cell lysates from non-targeting control or LC3B siRNA transfected HEY cells treated in the absence or presence of 5 μM MG132 were analyzed by western blotting with the indicated antibodies. Data shown is representative across three independent experiments. (**C**) Schematic summarizing the results presented herein. mRNA expression of NCOA4α and NCOA4β are increased in transformed endometriotic cells (compared to non-transformed controls) and malignant OVCA cell lines (compared to non-malignant gynecological cell lines). NCOA4α protein is increased in malignant OVCA cell lines (compared to non-malignant gynecological cell lines). In addition, total NCOA4 protein was increased in a subset of human OVCA tumors (compared to normal adjacent tissues). Modulation of NCOA4 expression in T80, HEY, and PE-A-OCV cells led to changes in cellular survival.

NCOA4 has been shown to be regulated in an iron-dependent manner by the E3 ubiquitin ligase HERC2 when intracellular iron levels increase [43]. To assess NCOA4 protein regulation, we treated our gynecological cells (T80, HEY, TOV21G, T-HESC, and PE-A-OCV) for 18 hours with MG132 (a proteasome inhibitor, at 5 μM) or cycloheximide (CHX, a translational inhibitor, at 2 mg/ml) in the absence or presence of FAC (250 μM). We observed that NCOA4 protein was reduced upon MG132 treatment in all cell lines, independent of FAC treatment suggesting that NCOA4 is regulated in a proteasome-independent manner (Figure 7A). Furthermore, NCOA4 expression was reduced upon CHX treatment in all cell lines assessed, independent of FAC treatment suggesting that translation is an important contributor to NCOA4 protein levels (Figure 7A). Since we also observed an increase in LC3B-II upon MG132 treatment (implicating increased autophagic flux [44]), we next investigated whether NCOA4 was degraded via the autophagic pathway. As shown in Figure 7B, LC3B knockdown in MG132 treated cells did not rescue NCOA4 protein expression suggesting an alternative mechanism of NCOA4 regulation.

## DISCUSSION

*In vitro* studies for the investigation of the transition from endometriosis to clear cell and/or endometrioid ovarian cancers has been challenging due to the limitation and availability of long-term cultures of endometriotic cells. Herein, we have successfully generated transformed endometriotic cells via overexpression of HRAS^V12A^, c-MYC^T58A^, along with p53 inactivation. These alterations represent those that occur early in the development of ovarian cancers. Specifically, in clear cell ovarian cancers, RAS mutations [45] and c-MYC amplifications [46] are prominent in addition to PIK3CA and PTEN alterations [47] which is expected to lead to increased tumorigenic potential associated with “iron addiction” [48]. Mutations in p53 are not commonly observed (only ∼10%) in ovarian clear cell carcinomas (OCCC) [49, 50], although one conflicting report suggests this is a common mutation in all OVCA subtypes [51]. Indeed, iron is a potent carcinogen that increases ROS causing cellular damage [5]. Endometriomas are characterized by elevated heme and iron content [6] which is thought to contribute to the transition to specific subtypes of ovarian cancers. Regulation of intracellular iron levels is mediated via various means including receptors (CD71 and FPN) that allow its import and export as well as storage molecules (ferritin) [12]. NCOA4 (represented by two well studied isoforms) is not only a recently identified molecule mediating regulation of intracellular iron content in a ferritinophagic pathway [28, 29] but also as a player in modulating hormone functions as a nuclear co-activator [15, 16, 32]. Other reported functions for NCOA4 include (a) regulation of DNA replication to modulate DNA stability [52], (b) modulation of DNA damage response and cellular senescence [53], and (c) increasing cellular proliferation and migration/invasion of breast as well as prostate cancer cells [17, 18]. Herein (as shown in Figure 7C), we have defined that NCOA4 is elevated in transformed endometriotic cells (relative to non-transformed cells), in malignant ovarian cancer cells and in a subset of ovarian tumors. Moreover, we have demonstrated that NCOA4 isoforms have the capacity to alter gynecological cell survival.

Transcriptional regulation of NCOA4 mediated by oncogenes has not been previously reported. Transformation of immortalized fallopian tube fimbriae with SV40 LTAg and c-MYC increases CD71 and decreases FPN protein [27]. Further, HRAS, c-MYC, and E1A are reported to regulate ferritin levels which leads to increased intracellular labile iron and proliferative indices [12, 24–26, 54]. We propose that the combination of HRAS, c-MYC, and p53 inactivation contributes to NCOA4 mRNA transcription. Further work is needed to identify potential c-MYC binding sites in the promoter region of NCOA4 as well as determining whether inhibition of the MAPK pathway could antagonize NCOA4 transcription or whether p53 inhibits NCOA4 expression. Although we identified that reduction of NCOA4 levels led to reduced cellular survival, we did not measure intracellular labile iron pool in our transformed endometriotic cells which would clarify whether it support cellular survival in an iron- and/or hormone-dependent manner. In this regard, our subcellular localization data placing NCOA4 in the cytoplasmic compartment for both isoforms implicates its role in both functions. This is in contrast to the NCOA4 localization in prostate cancer specimens in which the NCOA4α isoform is localized to the cytoplasmic compartment and the NCOA4β isoform is localized in the nuclear compartment [31].

From TCGA analyses, the DNA copy number of NCOA4 is not markedly altered compared to CD71 which is notably amplified (>25%). We propose that NCOA4 is instead dysregulated at the protein level as a result of altered HERC2 E3 ubiquitin ligase activity; indeed, HERC2 is mutated up to 20% in various cancers (obtained from cBioportal [55, 56]). While NCOA4 is known to be ubiquitinated by HERC2 (for proteasome degradation) in an iron-dependent manner [43], we found that proteasome inhibition with FAC treatment did not prevent NCOA4 protein degradation in our gynecological cells. Furthermore, NCOA4 protein remained reduced upon proteasome inhibition with autophagy inhibition (via siRNA-mediated knockdown of LC3B), suggesting that NCOA4 is regulated in a proteasome- and autophagy-independent manner in our gynecological cells. Our preliminary investigation to identify HERC2 mutations (which may inhibit its E3 ligase function) using the TCGA database [55, 56], uncovered a commonly mutated residue (R2126) which is expected to be “probably damaging” (assessed by PolyPhen-2 [57]) to the protein structure. Further investigations are needed to elucidate the mechanisms underlying NCOA4 protein dysregulation in ovarian cancer.

Since we identified a marked further increase in NCOA4β mRNA in NCOA4β overexpressing cells when maintained in –PR media (relative to +PR media), we propose that one or more factors contained within FBS may regulate NCOA4 expression; these factors may include lipophilic proteins including hormones, growth factors, and cytokines [58]. Indeed, NCOA4 is involved in regulating hormone responses (via its action as a nuclear receptor coactivator for androgen and estrogen receptors [16, 17, 32]) in addition to its novel role as a mediator of intracellular iron levels (as a ferritinophagic mediator [28, 29]); we propose that NCOA4 may be involved in OVCA biology by both mechanisms of action. Prior data identifies links between estrogen/androgen levels (hormones) and iron signaling; for example, there is an inverse relationship between estrogen and body iron levels in post-menopausal women [59]. Furthermore, estrogen is well-established to regulate FPN by binding to estrogen response elements (ERE) within its promoter region [60]. Estrogen is an important contributor to ovarian cancer development [61] and can itself modulate p53 expression [62] which transcriptionally regulates ISCU expression leading to changes in ferritin and CD71 levels [63]. However, further investigations are needed to fully elucidate the roles of NCOA4α and NCOA4β in OVCA biology as well as their response to –PR media conditions.

Our findings support a need for further investigations on regulatory mechanisms underlying NCOA4 isoform expression and function in specific subtypes of ovarian cancers. To this end, developing a combination NCOA4 knockout mice [29] with an OVCA mouse model [64] may provide insight into the role of NCOA4 in OVCA biology.

## MATERIALS AND METHODS

### Ethics and human OVCA tissue microarray

We obtained a TMA (US Biomax, Inc. (#BC11115b, Derwood, MD)) containing a total of 100 core samples including 5 clear cell carcinomas, 48 serous papillary adenocarcinomas, 14 serous adenocarcinomas, 12 mucinous adenocarcinomas, 1 endometrioid adenocarcinoma, 10 lymph node metastasis adenocarcinoma, and 10 adjacent normal tissues which were de-identified and cannot be linked to the human subjects. The studies that were performed using this array as reported in this manuscript received an assessment of Not Human Subjects Research Determination (IRB#: Pro00029616) from the University of South Florida IRB Review Board whose Chairperson is Dr. Jorgensen. The TMA was stained using previously published methods [65] with a 1:100 dilution of the NCOA4 antibody (Novus Biologicals, #H00008031-M05) for a 45 minute incubation with an epitope retrieval solution 2 for 20 minutes. The stained TMA was scanned using the Aperio ScanScope XT (Aperio Vista, CA, USA) as described previously [65]. Representative images were captured at 6× magnification. Positive core analyses were determined using the Positive Pixel Count v.9.0 algorithm. Thresholds are as follows: Hue Value (Center) = 0.1; Hue Width = 0.5; Color Saturation Threshold = 0.04; Intensity Threshold Weak (Upper Limit) = 220; Intensity Threshold Weak (Lower Limit) = 175; Intensity Threshold Medium (Upper Limit) = 175; Intensity Threshold Medium (Lower Limit) = 100; Intensity Threshold Strong (Upper Limit) = 100; Intensity Threshold Strong (Lower Limit) = 0. Statistical Analysis (student's nonparametric *t*-test) was completed in GraphPad Prism, and error bars represent the mean ± SD ^*^p < 0.05, ^**^p < 0.01.

### Cell culture

Human normal immortalized (with LTAg and hTERT) ovarian surface epithelial T80 cells and serous ovarian epithelial HEY carcinoma cells were kindly provided by Dr. Gordon Mills (MD Anderson Cancer Center, Houston, TX, USA) and were maintained in RPMI 1640 supplemented with 8% FBS and 1% penicillin/streptomycin as previously described [33]. Human immortalized (with hTERT) endometrial stromal T-HESC cells were obtained from ATCC (Manassas, VA, USA) and were maintained in phenol-red free DMEM/F12 (1:1) supplemented with 8% charcoal-dextran treated FBS, 500 ng/ml puromycin, 1% ITS+ Premix (#354352, BD Biosciences, San Jose, CA, USA), and 15 mM HEPES as previously described [65]. Human clear cell ovarian TOV21G carcinoma cells were kindly provided by Dr. Jonathan Lancaster (Moffitt Cancer Center, Tampa, FL, USA) while human endometrioid ovarian TOV112D carcinoma cells were obtained from ATCC (Manassas, VA, USA); both cell lines were maintained in MCDB 131/Medium 199 (1:1) supplemented with 8% FBS and 1% penicillin/streptomycin. Fallopian tube secretory epithelial cells (FTSEC (FT194)) were kindly provided by Dr. Ronald Drapkin (University of Pennsylvania, Philadelphia, PA, USA) and maintained in DMEM/F12 (1:1) supplemented with 2% Ultroser G (Crescent Chemical Company, #67042.1/S) and 1% penicillin/streptomycin. T80, T-HESC, HEY, TOV21G, and TOV112D were authenticated by STR profiling (Genetica Laboratories, Cincinnati, OH, USA). The STR profiles for human endometriotic cells were confirmed to not match any profiles representing commercial cell lines deposited within the repository cell line database (Genetica Laboratories, Cincinnati, OH, USA). Furthermore, all cell lines were confirmed to be mycoplasma negative.

### Human primary endometriotic cell isolation

Human primary endometriotic cell lines (kindly provided by Dr. Idhaliz Flores, Ponce, Puerto Rico) were obtained from four endometriotic lesions as follows: (a) PE-A cells, derived from a lesion in the posterior cul-de-sac, (b) PE-B cells, derived from a lesion in the right mesosalpinx, (c) PE-C cells, derived from a peritoneal lesion, and (d) PE-D cells, derived from an endometrioma located on the right ovary. These cells were maintained in MCDB 131/Medium 199 (1:1) supplemented with 8% FBS and 1% penicillin/streptomycin.

### Generation of human transformed endometriotic cells

HEK293T packaging cells were seeded at 1.5×10^6^ cells in 6-well plates. Following overnight adherence, cells were transfected with a retroviral expression plasmid concurrently with pCGP and pVSVG plasmids (1:1:1 ratio) using Fugene HD (Promega, Madison, MI, USA). Control virus was generated using an empty pBABE-puro expression plasmid (Addgene plasmid #1764 [66]). Oncogenic cocktail virus was generated using the following at equimolar quantities (a) H-RAS^V12A^ (Addgene plasmid #9051, a gift from William Hahn), (b) c-MYC^T58A^ (Addgene plasmid #20076, a gift from Juan Belmonte [67]), (c) SV40 LTAg (Addgene plasmid #14088, a gift from William Hahn [68]), and (d) HA-tagged hTERT (Addgene plasmid #1772, a gift from Bob Weinberg [69]). Forty-eight hours after transfection, media supernatant (containing retrovirions) was collected, filtered (0.45μm), and used to infect PE-A, PE-B, PE-C, and PE-D human primary endometriotic cells with 8 μg/ml polybrene. Following the initial retroviral infection, cells were maintained for 23 days at which time there was no further increase in cell growth in either the OCV (oncogenic virus cocktail) or CV (control virus) infected cells. Thus, two additional rounds of retroviral infections were completed and cells were maintained for 44 days, at which time both the PE-C and PE-D (CV and OCV infected cells) reached crisis. PE-A and PE-B control virus infected cells also eventually reached crisis, at which point the OCV infected cells, which continued to survive and proliferate, were considered to be transformed. We completed two additional rounds of infections in PE-A and PE-B cells (with CV and OCV retrovirions) to increase cell numbers available for analyses since the number of non-transformed cells was a limitation for the subsequent analyses.

### Cloning of NCOA4α and NCOA4β into a retroviral expression plasmid for generation of retroviral cell lines

RNA isolated from T80 cells using an RNeasy kit (QIAGEN, Germantown, MD, USA) was utilized for reverse transcriptase (RT)-polymerase chain reaction (PCR) to amplify the full-length form of NCOA4 (NCOA4α). RNA isolated from TOV21G cells using an RNeasy kit (QIAGEN, Germantown, MD, USA) was utilized for reverse transcriptase (RT)-polymerase chain reaction (PCR) to amplify the shorter form of NCOA4 (NCOA4β). The primers used are (1) 5′-GGG-GAATTC-ACCGCC-ATG-AAT-ACC-TTC-CAA-GAC-3′ (forward primer) and (2) 5′-GGG-GAATTC-TCA-CAT-CTG-TAG-AGG-AGT-TCG-3′ (reverse primer). The PCR conditions used were as follows: 30 min at 48°C, 2 min at 94°C followed by 40 cycles of 1 min at 94°C, 1 min at 55°C (for both NCOA4α and NCOA4β), 5 min at 68°C. The final extension step was set for 15 min at 72°C. Products were analyzed on a 1% agarose gel and the appropriately sized PCR products for both NCOA4α and NCOA4β were gel purified using the QIAquick Gel Extraction kit (QIAGEN, Germantown, MD, USA) following the manufacturer's protocol and then cloned into the pTOPO vector (Invitrogen, Carlsbad, CA, USA). Positive clones were validated by sequencing (Moffitt Cancer Center, Molecular Genomics Core, Tampa, Florida, USA). NCOA4α and NCOA4β were then subcloned into the EcoRI site in the retroviral pBABE-puro (Addgene plasmid #1764[66]) and pQCXIN (Clontech, Mountain View, CA, USA) expression vectors. Large plasmid stocks of NCOA4α and NCOA4β in these retroviral plasmids were generated using the EndoFree Plasmid kit (QIAGEN, Germantown, MD, USA).

HEK293T packaging cells were transfected with the appropriate expression vector (pBABE-puro or pQCXIN) or the corresponding empty vector (as control) as well as the pCGP and pVSVG vectors (at a 1:1:1 ratio). At 48 and 72 hours post-transfection, virus-containing supernatant was collected, filtered (0.45 μm), and used to infect the appropriate target cells with 8 μg/ml polybrene (first round of infection) or 16 μg/ml polybrene (second round of infection). Cells were then expanded to T75 flasks in appropriate antibiotic-containing media (1 μg/ml puromycin for T80 cells, 2 mg/ml puromycin for HEY cells, and 0.5 μg/ml G418 for PE-A-OCV cells).

### Cellular treatments

Media from senescent PE-A-CV and PE-B-CV cells was collected (conditioned media, COM), 0.22 mm filtered, and stored at −20°C until use in migration assays. Ferric ammonium citrate (FAC) was obtained from Fisher Scientific (Pittsburgh, PA, USA); a 50 mM stock was prepared in PBS and used at a final concentration of 250 μM according to our prior work [33]. To prepare serum factor-depleted media (-PR), phenol-red free DMEM/F12 (1:1) was supplemented with 8% charcoal-dextran treated FBS and 1% penicillin/streptomycin. MG132 (Fisher Scientific, Pittsburgh, PA, USA), a proteasome inhibitor, was prepared in dimethyl sulfoxide (DMSO) at 10 mM and used at a final concentration of 5 μM while cycloheximide (CHX, Fisher Scientific, Pittsburgh, PA, USA), a protein synthesis inhibitor, was prepared in DMSO at 1 mg/ml and used at a final concentration of 2 μg/ml as previously described [70] in the presence or absence of 250 μM FAC for 18 hours.

### siRNA transfection

Cells were seeded at 350,000 cells (T80, T-HESC, HEY, PE-A-OCV) or 1×10^6^ cells (TOV21G) per well in 6-well plates and allowed to adhere overnight. Cells were then transfected with the appropriate siRNA using DharmaFECT 1 (Fisher Scientific, Pittsburgh, PA, USA) according to our previously published methods [71]. The following ON-TARGETplus siRNAs were obtained from GE Dharmacon (Lafayette, CO, USA): (1) non-targeting control (D-001810-10-20), (2) NCOA4 (L-010321-00-0005), and (3) LC3B (L-012846-00-0005). To obtain efficient knockdown of NCOA4, two rounds of siRNA transfection were performed while one round was completed for LC3B.

### RNA Isolation and real-time polymerase chain reaction (PCR)

The RNeasy Kit (QIAGEN, Valencia, CA, USA) was used to isolated total RNA following the manufacturer's instructions. Using methods previously described [33], real-time PCR was performed using the One-step Master Mix from Applied Biosystems (#4392938, Foster City, CA, USA) with the following FAM-labelled probes/primers: (1) NCOA4 (Hs00428331_g1), (2) FTH (Hs01694011_s1), (3) CD71 (Hs00951083_m1), (4) FPN (Hs00205888_m1), (5) DMT1 (Hs00167206_m1), (6) ISCU (Hs00384510_m1), (7) IL-6 (Hs00985639_m1), (8) SNAIL (Hs00195591_m1), (9) SLUG (Hs00950344_m1), (10) ZEB1 (Hs00232783_m1), (11) ZEB2 (Hs00207691_m1), (12) TWIST1 (Hs00361186-m1), (13) LC3B (Hs00797944-s1), (14) LAMP1 (Hs00174766-m1), (15) SQSTM1 (Hs01061917-g1), and (16) MCOLN1 (Hs01100653-m1). Using the Custom TaqMan^®^ Assay Design Tool, FAM-labelled probes and primers specific for NCOA4α were designed to cross the junction between exon 8 and exon 9 while FAM-labelled probes and primers specific for NCOA4β were designed to cross the junction between exon 7 and exon 9. C_T_ values were normalized to β-actin (#401846, Applied Biosystems, Foster City, CA, USA) and RNA-fold changes were calculated using the formula 2^−ΔΔCT^.

### Protein isolation, SDS-PAGE, and western analyses

Protein lysates were prepared according to previously published methods [72]. Proteins were then normalized and loaded onto appropriate (10%) SDS-PAGE gels which were next transferred onto polyvinylidene fluoride (PVDF) membranes. As indicated in the Figure legends, samples were re-run to prevent overlapping detection of similar molecular weight antigens. Western blotting was conducted according to previously described methods [71]. Total Ras rabbit monoclonal (#3339, 1:1000), FTH rabbit polyclonal (#3998, 1:500), c-MYC rabbit monoclonal (#13987, 1:500), LC3B rabbit polyclonal (#2775, 1:1000), p21 mouse monoclonal (#2946, 1:250), phospho-p42/44 MAPK rabbit polyclonal (#9101, 1:750), pAKT rabbit monoclonal (#4060, 1:1000), pan-AKT rabbit monoclonal (#4685, 1:1000), Lamin A/C mouse monoclonal (#4777, 1:2000), PARP rabbit polyclonal (#9542, 1:1000), GAPDH rabbit monoclonal (#5174, 1:1000), and pan-Actin rabbit polyclonal (#4968, 1:500) antibodies were obtained from Cell Signaling Technology (Danvers, MA, USA). SV40 Large T antigen mouse monoclonal (#554149, 1:1000) and p62 mouse monoclonal (#610832, 1:1000) were obtained from BD Biosciences (San Jose, CA, USA). NCOA4 rabbit polyclonal (#A302–272a, 1:1000) was obtained from Bethyl Laboratories (Montgomery, TX, USA). CD71 mouse monoclonal (#sc-51829, 1:250) was obtained from Santa Cruz Biotechnology (Dallas, TX, USA). ID1 rabbit antibody (1:300) was kindly provided by Dr. Miguel Pujana (Catalan Institute of Oncology, Barcelona, Spain). Densitometric analyses were completed using ImageJ (NIH).

### Immunofluorescence

Human endometriotic cells (PE-A-CV and PE-A-OCV as well as PE-B-CV and PE-B-OCV) were seeded onto glass coverslips and grown until sufficient numbers of cells were present prior to staining. Cells were fixed for 30 minutes in 4% formaldehyde and then blocked (5% goat serum and 0.1% Triton X-100 in PBS) for 1 hour at room temperature. Rabbit monoclonal antibody targeting γH2AX was applied at 1:400 dilution overnight at 4°C (#9718, Cell Signaling Technology) in a humidifying chamber. The following day, the cells were incubated with the appropriate secondary antibody (Alexa Fluor 488 goat anti-rabbit used at 1:500 dilution in 1% goat serum and 0.1% Triton-X-100 in PBS) for 1 hour (in the dark).

For staining of actin filaments, cells grown on coverslips were fixed and blocked as described above. Cells were then incubated at room temperature for 30 minutes with 5 units of Alexa Fluor 488 Phalloidin (#A12379, Fisher Scientific) according to manufacturer's guidelines. Slides were viewed and imaged at 63× and 20× magnification using a PerkinElmer UltraVIEW Confocal spinning disc microscope (PerkinElmer Corporation).

### Subcellular fractionation

Cytoplasmic and nuclear fractions were obtained using the Subcellular Protein Fractionation Kit (#78840, Fisher Scientific) according to manufacturer's instructions. The separated fractions were then run on appropriate SDS-PAGE gels and analyzed by western blotting.

### Cell viability assays

Cells were seeded at 2,500 cells per well in a 96-well opaque plate. After 72 hours, the CellTiter-Glo Luminescent Cell Viability Assay (Promega, Madison, WI, USA) was performed according to manufacturer's instructions. To assess changes in viability upon NCOA4 siRNA, cells (T80, HEY, and PE-A-OCV) were re-seeded following the last round of transfection with siRNA. The following day, wells were rinsed with PBS before incubating the cells in serum factor-replete (+PR), serum factor-deplete (-PR), or FAC (250 μM) media for 24 hours. For control (empty vector) and NCOA4β overexpressing cells, cell viability was assessed following 72 hours post-seeding.

### Migration assay

The migration assay was performed according to manufacturer's protocol (#CBA-110, Cell Biolabs, San Diego, CA, USA). Briefly, PE-A and PE-B (infected with CV and OCV) cells were seeded in 300 ml serum-free media at 15,000 cells/well in Boyden chamber inserts within a 24-well plate. Complete (serum-containing) media (CM), as chemoattractant, was added (500 ml) to the bottom chamber of the well. After a 24 hour incubation, the upper chamber of the Boyden chamber insert was carefully cleaned using Q-tips and then stained for 10 minutes with 400 ml of the provided Cell Stain Solution. Following extensive washing, representative light microscope images were captured at 100× magnification. Furthermore, cells were counted twice independently to quantify migrated cells. For treatment with senescence-conditioned media (COM), PE-A-OCV and PE-B-OCV cells were seeded similarly to that described above and complete (CM, as control) or COM media were added as the chemoattractant to the bottom chamber. Following 24 hour incubation, cells were stained and images captured as described above.

### 3D morphogenesis assay

Using published methods for sphere formation [73], 100% Matrigel Basement Membrane Matrix (#356234, Fisher Scientific, Pittsburgh, PA, USA) was thawed on ice (in a 4°C refrigerator) overnight prior to use. The following day, 40 ml of the stock Matrigel was added to each chamber of an 8-well Chamber Slide (#PEZGS0816, Fisher Scientific) using pre-chilled pipet tips; this was then incubated at 37°C for 15 minutes to solidify. PE-A and PE-B (CV and OCV) cells were next seeded at 5,000 cells in a total volume of 400 ml (containing a final concentration of 2% Matrigel in complete media) per chamber. Cells were re-fed every 4 days by removing the top layer of media and overlaying with fresh complete media containing 2% Matrigel. On the 10^th^ day, representative light microscope images were captured at 100× and 200× magnification (images were converted to grayscale in PowerPoint).

### Senescence assay

Senescent cells were identified by using the Senescence β-Galactosidase Staining Kit (#9860, Cell Signaling Technology) according to manufacturer's instructions. Representative images were captured using a light microscope at 100× magnification.

### Colony formation assay

Cells were seeded at 500 cells per well in 6-well plates and allowed to grow for the times indicated in the Figure Legends. The plates were then stained with crystal violet and scanned.

### Statistical analyses

All analyses were performed using GraphPad version 6.04 Prism software (GraphPad, La Jolla, CA, USA) and *p*-values were calculated using the non-parametric Student's *t*-test. The displayed error bars represent the mean ± SD. NS represents non-significant *p*-values; * represents *p*-values ≤ 0.05; ** represents *p*-values ≤ 0.01; *** represents *p*-values ≤ 0.001; and **** represents *p*-values ≤ 0.0001. Where indicated, fold changes and percent reductions were calculated as an average of three independent replicates.

## SUPPLEMENTARY FIGURES



## Abbreviations

AKT: AKT Serine/Threonine Kinase 1
CD71: Transferrin Receptor
c-MYC: Chicken Myelocytomatosis
DMT1: Divalent Metal Transporter 1
EMT: Epithelial-to-Mesenchymal Transition
FTH1: Ferritin Heavy Chain 1
FPN: Ferroportin
HRAS: Harvey Rat Sarcoma Viral Oncogene Homolog
HERC2: HECT and RLD Domain Containing E3 Ubiquitin Protein Ligase 2
hTERT: human telomerase reverse transcriptase
IL-6: Interleukin 6
ISCU: Iron-Sulfur Cluster Scaffold Protein
LAMP1: Lysosomal Associated Membrane Protein 1
LC3B: Microtubule Associated Protein 1 Light Chain 3 Beta
MAPK: Mitogen-Activated Protein Kinase 1
MCOLN1: Mucolipin 1
NCOA4: Nuclear Receptor Coactivator 4
Rb: Retinoblastoma
SASP: Senescence-Associated Secretory Phenotype
siRNA: small interfering RNA
SNAIL: Snail Family Transcriptional Repressor 1
SLUG: Snail Family Transcriptional Repressor 2
SQSTM1: Sequestosome
SV40 LTAg: Simian Vacuolating Virus 40 Large-T Antigen
TMA: Tissue Microarray
TWIST: Twist Family BHLH Transcription Factor 1
ZEB1: Zinc Finger E-Box Binding Homeobox 1
ZEB2: Zinc Finger E-Box Binding Homeobox 2
