# Supplementary material for: Expression and function of nuclear receptor coactivator 4 isoforms in transformed endometriotic and malignant ovarian cells
Source: Oncotarget. 2017 Dec 28;9(4):5344–67. doi: 10.18632/oncotarget.23747 (PMC5797054; doi:10.18632/oncotarget.23747)
Supplement: Supplementary file 1 [file oncotarget-09-5344-s001.pdf]

Expression and function of nuclear receptor coactivator 4 isoforms in transformed endometriotic and malignant ovarian cells

SUPPLEMENTARY MATERIALS

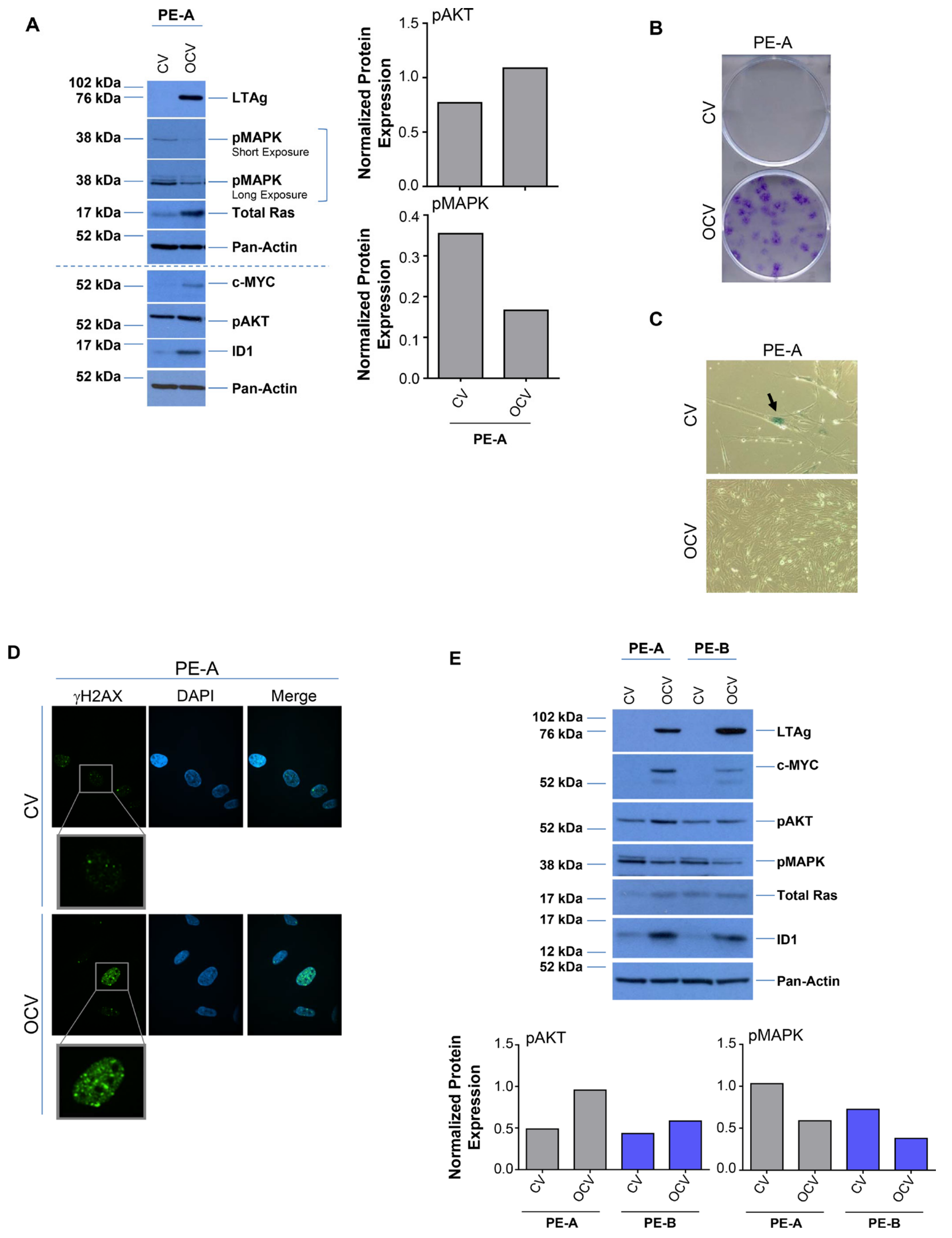

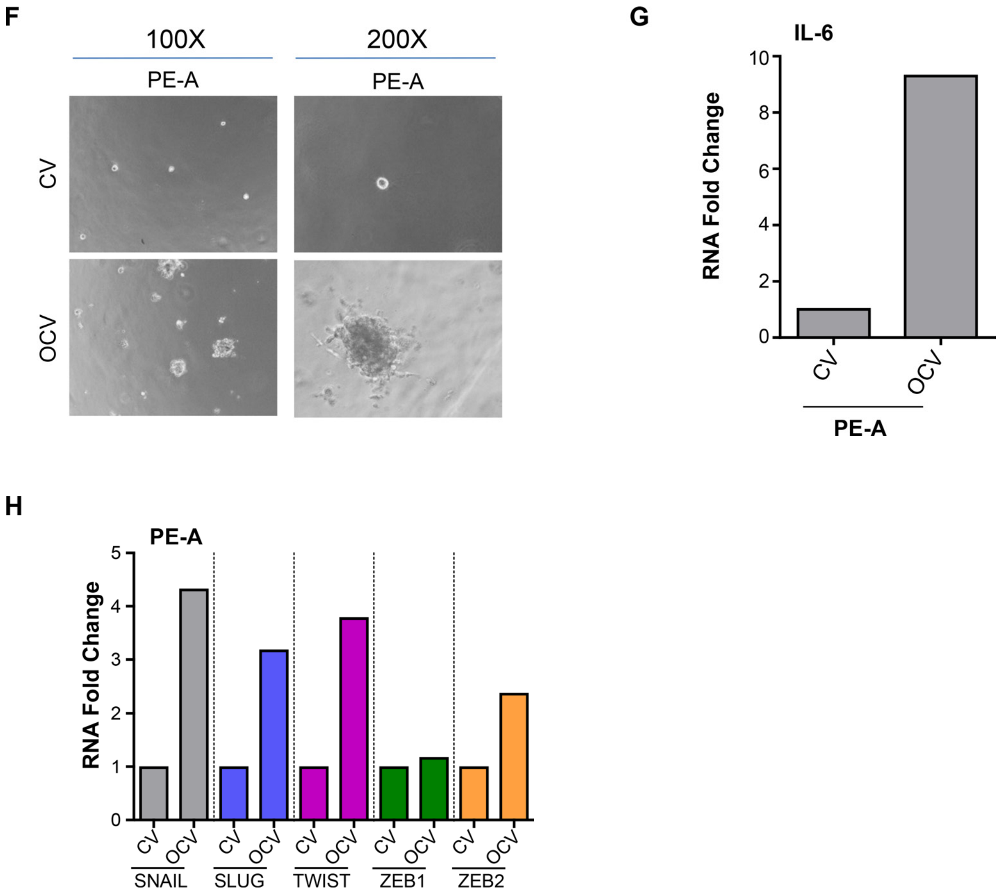

Supplementary Figure 1: Cellular transformation of human primary endometriotic cells. The first batch of retrovirally infected cells (PE-A-CV and PE-A-OCV) were utilized to: (**A**) obtain cell lysates for western blotting with the indicated antibodies (left panel). The dotted line specifies re-run samples to avoid the possibility of detecting overlapping bands of similar molecular weights. Densitometric analyses for pAKT and pMAPK are shown in the right panels; (**B**) perform colony formation assay and images were captured following 14 days in culture (representative images are shown, three independent experiments were conducted); (**C**) perform β-galactosidase staining and images were captured at 100X magnification (representative images are shown, three independent experiments were conducted); and (**D**) assess DNA damage via γH2AX immunofluorescence staining (representative images shown were captured at 63× magnification and the images of nuclei were enlarged and cropped using PowerPoint to focus on the DNA damage foci). The second batch of retrovirally infected cells (PE-A-CV and PE-B-CV as well as PE-A-OCV and PE-B-OCV) were utilized to: (**E**) obtain cell lysates for western blotting with the indicated antibodies (left panel). Densitometric analyses for pAKT and pMAPK are shown in the bottom panels; (**F**) assess the *in vitro* tumorigenic potential (by 3-dimensional morphogenesis assay). Representative images (from four independent experiments) were captured at 100× (left) and 200X (right) magnification. The second batch of retrovirally infected cells (PE-A-CV and PE-A-OCV) were utilized to: (**G**) to measure IL-6 transcript levels via real-time PCR. Three independent experiments were performed; and (**H**) assess transcript levels for genes in the EMT pathway via real-time PCR (data shown are from one independent experiment (due to limiting cell numbers in PE-A-CV cells)).

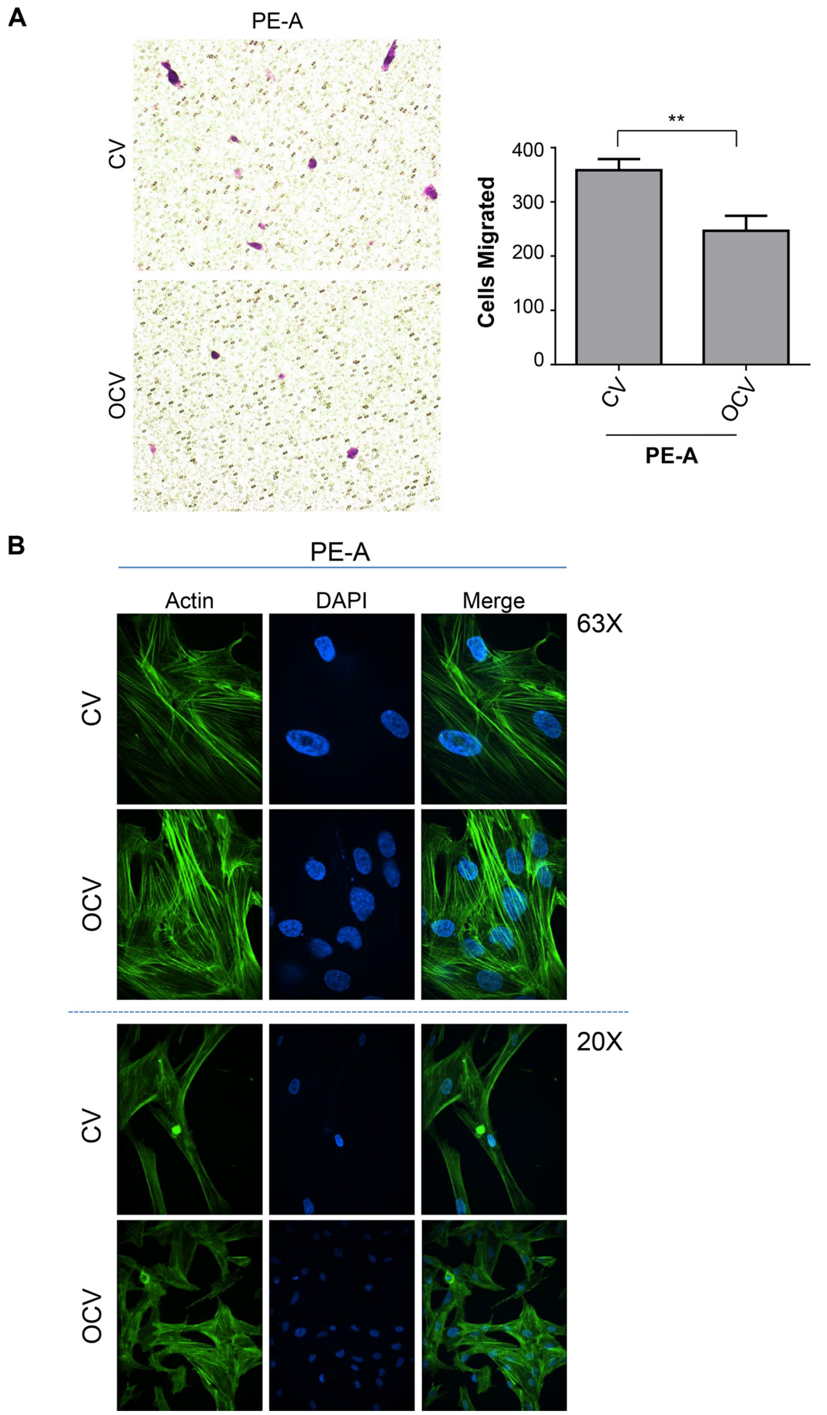

Supplementary Figure 2: Conditioned media from senescent primary endometriotic cells promotes migration of transformed endometriotic cells. The second batch of retrovirally infected cells (PE-A-CV and PE-A-OCV) were utilized to: (**A**) perform migration assay. Representative images (from four independent experiments) were captured at 100× magnification (left panel). Manual cell counts are presented in the right panel; and (**B**) assess actin filament organization using phalloidin staining. Representative images (from three independent experiments) are shown at 63X (top panel) and 20X (bottom panel) magnification.

A

```
NCOA4-Alpha    1    MNTFQDQSGS SSNREPLLRC SDARRDLELA IGGVLRAEQQ IKDNLREVKA
NCOA4-Beta     1    MNTFQDQSGS SSNREPLLRC SDARRDLELA IGGVLRAEQQ IKDNLREVKA

NCOA4-Alpha   51    QIHSCISRHL ECLRSREVWL YEQVDLIYQL KEETLQQQAQ QLYSLLGQFN
NCOA4-Beta    51    QIHSCISRHL ECLRSREVWL YEQVDLIYQL KEETLQQQAQ QLYSLLGQFN

NCOA4-Alpha  101    CLTHQLECTQ NKDLANQVSV CLERLGSLTL KPEDSTVLLF EADTITLRQT
NCOA4-Beta   101    CLTHQLECTQ NKDLANQVSV CLERLGSLTL KPEDSTVLLF EADTITLRQT

NCOA4-Alpha  151    ITTFGSLKTI QIPEHLMAHA SSANIGPFLE KRGCISMPEQ KSASGIVAVP
NCOA4-Beta   151    ITTFGSLKTI QIPEHLMAHA SSANIGPFLE KRGCISMPEQ KSASGIVAVP

NCOA4-Alpha  201    FSEWLLGSKP ASGYQAPYIP STDPQDWLTQ KQTLENSQTS SRACNFFNNV
NCOA4-Beta   201    FSEWLLGSKP ASGYQAPYIP STDPQDWLTQ KQTLENS--- ----------

NCOA4-Alpha  251    GGNLKGLENW LLKSEKSSYQ KCNSHSTTSS FSIEMEKVGD QELPDQDEMD
NCOA4-Beta   238    ---------- ---------- ---------- ---------- ----------

NCOA4-Alpha  301    LSDWLVTPQE SHKLRKPENG SRETSEKFKL LFQSYNVNDW LVKTDSCTNC
NCOA4-Beta   238    ---------- ---------- ---------- ---------- ----------

NCOA4-Alpha  351    QGNQPKGVEI ENLGNLKCLN DHLEAKKPLS TPSMVTEDWL VQNHQDPCKV
NCOA4-Beta   238    ---------- ---------- ---------- ---------- ----------

NCOA4-Alpha  401    EEVCRANEPC TSFAECVCDE NCEKEALYKW LLKKEGKDKN GMPVEPKPEP
NCOA4-Beta   238    ---------- ---------- ---------- ---------- ----------

NCOA4-Alpha  451    EKHKDSLNMW LCPRKEVIEQ TKAPKAMTPS RIADSFQVIK NSPLSEWLIR
NCOA4-Beta   238    ---------- ---------- ---------- ---------- ----------

NCOA4-Alpha  501    PPYKEGSPKE VPGTEDRAGK QKFKSPMNTS WCSFNTADWV LPGKKMGNLS
NCOA4-Beta   238    ---------- ---------- ---------- ---------- ----------

NCOA4-Alpha  551    QLSSGEDKWL LRKKAQEVLL NSPLQEEHNF PPDHYGLPAV CDLFACMQLK
NCOA4-Beta   238    ---------- -----QEVLL NSPLQEEHNF PPDHYGLPAV CDLFACMQLK

NCOA4-Alpha  601    VDKEKWLYRT PLQM
NCOA4-Beta   273    VDKEKWLYRT PLQM
```

AR Binding Motif (LYSLL; FKL LF)

Oligomerization Domain

AhR interacting Domain

Ferritin Binding Domain

AhR interacting Domain

B

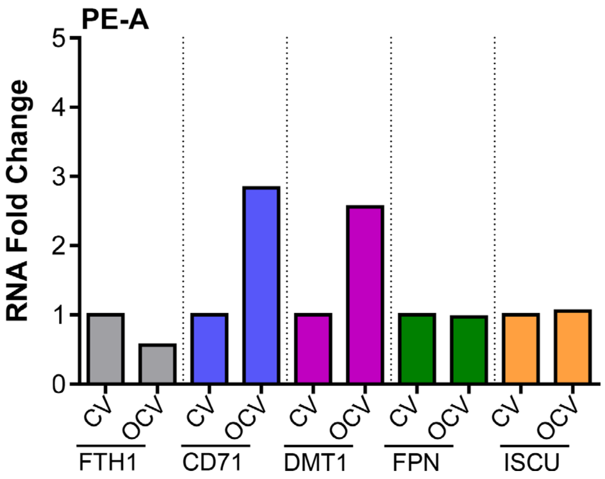

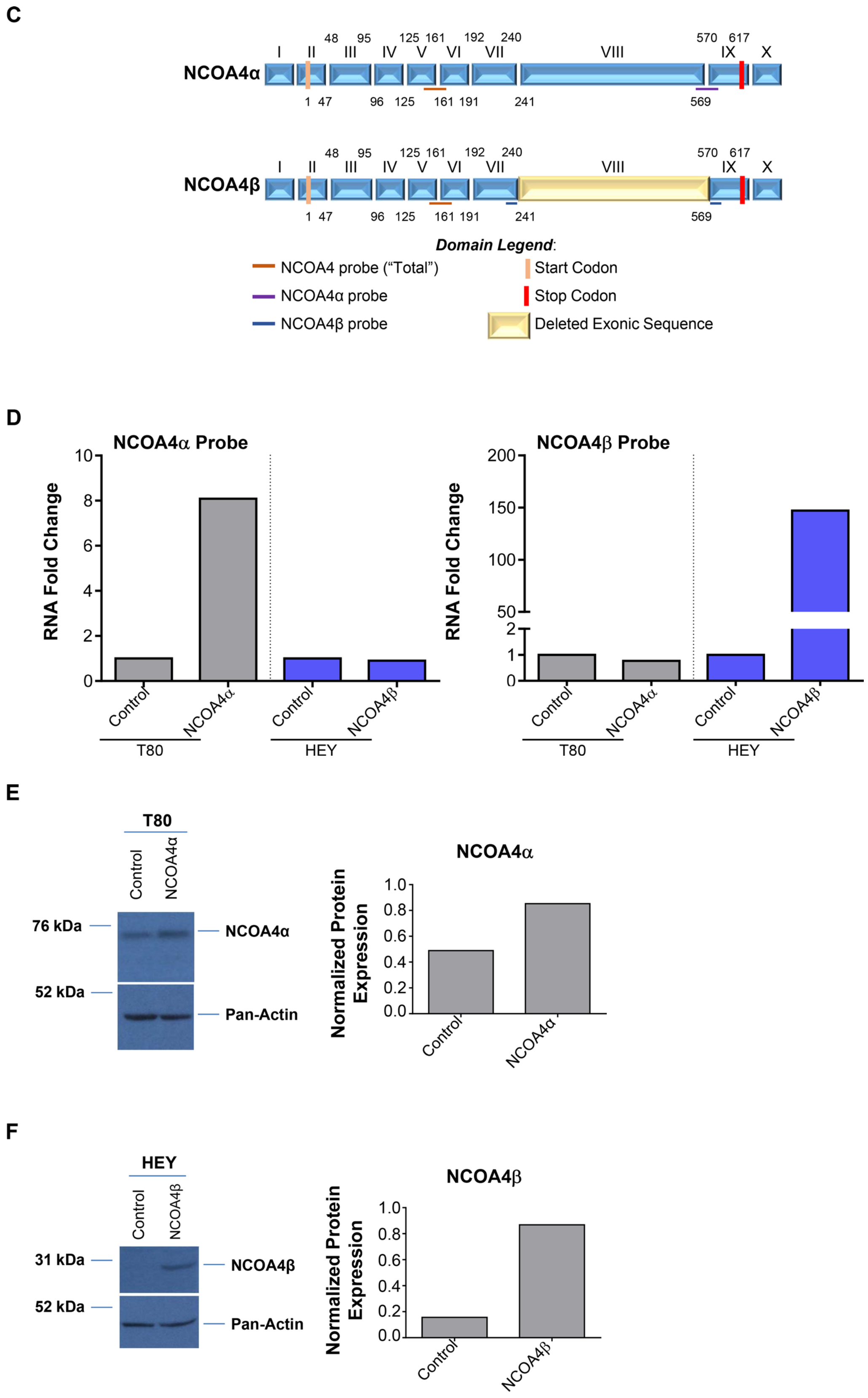
C
NCOA4α
NCOA4β
I II III IV V VI VII VIII IX X
48 95 125 161 192 240 570 617
1 47 96 125 161 191 241 569
Domain Legend:
NCOA4 probe ("Total")
NCOA4α probe
NCOA4β probe
Start Codon
Stop Codon
Deleted Exonic Sequence
D
NCOA4α Probe
NCOA4β Probe
RNA Fold Change
Control
NCOA4α
NCOA4β
T80
HEY
E
T80
Control
NCOA4α
76 kDa
52 kDa
Pan-Actin
Normalized Protein Expression
F
HEY
NCOA4β
31 kDa
52 kDa
Pan-Actin
Normalized Protein Expression

G

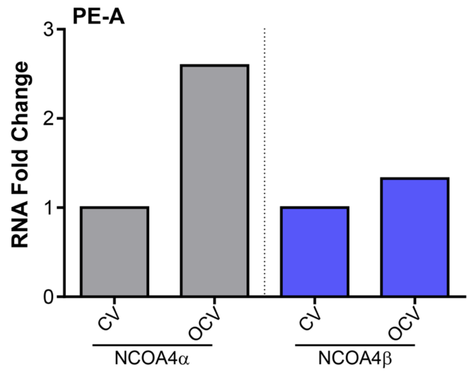

H

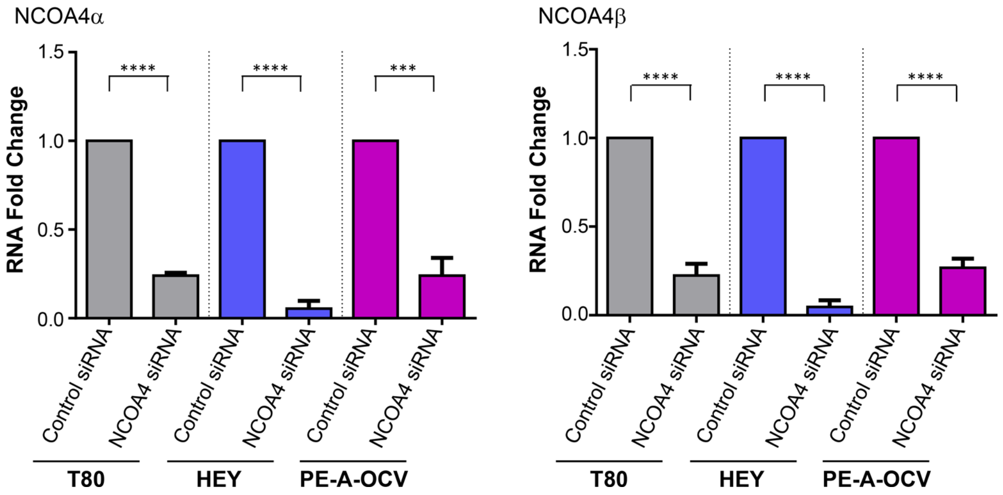

Supplementary Figure 3: Validation of real-time PCR probes/primers for NCOA4 isoforms and their increased transcript expression in transformed endometriotic cells. (**A**) Using DiAlign software (Genomatix), the protein sequence from our NCOA4β clone was aligned with NCOA4α (accession #: Q13772). Dashes represent internally deleted sequence in the NCOA4β variant while highlighted regions represent defined binding domains (i.e., oligomerization domain (amino acid 17 to 125), two AhR interaction domains (amino acids 235 to 321 and amino acids 441 to 556), ferritin binding domain (amino acids 383 to 522), and two androgen receptor binding motifs (amino acids 92 to 328). (**B**) Using RNA extracted from the second batch of PE-A-CV and PE-A-OCV cells, transcript levels for genes in the iron pathway were assessed via real-time PCR. Data shown are from one independent experiment (due to limiting cell numbers in PE-A-CV cells). (**C**) The location of the probes to detect total NCOA4 (Assays-on-Demand) and NCOA4α and NCOA4β (Assays-by-Design) are shown. (**D**) RNA isolated from NCOA4α overexpressing T80 and NCOA4β overexpressing HEY cells was utilized to validate the available NCOA4 probe/primer sets. (**E**) Cell lysates from control and NCOA4α overexpressing T80 cells were analyzed by western blotting with the indicated antibodies (left panel). Densitometric analysis is presented (right panel). (**F**) Cell lysates from control and NCOA4β overexpressing HEY cells were analyzed by western blotting with the indicated antibodies (left panel). Densitometric analysis for NCOA4β is presented (right panel). (**G**) RNA from the second batch of PE-A-CV and PE-A-OCV cells was utilized to assess transcript levels of NCOA4α and NCOA4β via real-time PCR. Data shown are from one independent experiment (due to limiting cell numbers in PE-A-CV cells). (**H**) T80, HEY, and second batch of PE-A-OCV cells were treated with control or NCOA4 siRNA. Isolated RNA was utilized to assess transcript levels of NCOA4α and NCOA4β via real-time PCR. Three independent experiments were performed.
